# Long non-coding RNA exploration for mesenchymal stem cell characterisation

**DOI:** 10.1186/s12864-020-07289-0

**Published:** 2021-06-04

**Authors:** Sébastien Riquier, Marc Mathieu, Chloé Bessiere, Anthony Boureux, Florence Ruffle, Jean-Marc Lemaitre, Farida Djouad, Nicolas Gilbert, Thérèse Commes

**Affiliations:** IRMB, University of Montpellier, INSERM, 80 rue Augustin Fliche, Montpellier, France

**Keywords:** Mesenchymal stem cell, Transcriptomics, Long non-coding RNA, RNAseq, NGS analysis, Bioinformatics

## Abstract

**Background:**

The development of RNA sequencing (RNAseq) and the corresponding emergence of public datasets have created new avenues of transcriptional marker search. The long non-coding RNAs (lncRNAs) constitute an emerging class of transcripts with a potential for high tissue specificity and function. Therefore, we tested the biomarker potential of lncRNAs on Mesenchymal Stem Cells (MSCs), a complex type of adult multipotent stem cells of diverse tissue origins, that is frequently used in clinics but which is lacking extensive characterization.

**Results:**

We developed a dedicated bioinformatics pipeline for the purpose of building a cell-specific catalogue of unannotated lncRNAs. The pipeline performs ab initio transcript identification, pseudoalignment and uses new methodologies such as a specific k-mer approach for naive quantification of expression in numerous RNAseq data. We next applied it on MSCs, and our pipeline was able to highlight novel lncRNAs with high cell specificity. Furthermore, with original and efficient approaches for functional prediction, we demonstrated that each candidate represents one specific state of MSCs biology.

**Conclusions:**

We showed that our approach can be employed to harness lncRNAs as cell markers. More specifically, our results suggest different candidates as potential actors in MSCs biology and propose promising directions for future experimental investigations.

**Supplementary Information:**

The online version contains supplementary material available at (10.1186/s12864-020-07289-0).

## Background

The increasing popularity of RNAseq and the ensuing aggregation of this type of data into public databases enable the search for new biomarkers across large cohorts of donors or cell types for the identification of pathological conditions or cellular lineages. As such, RNAseq has paved the way for the discovery of novel transcriptional biomarkers such as long non-coding RNAs (lncRNAs), that have emerged as a fundamental molecular class. A growing number of lncRNAs has been identified in the last decades, with their number approaching that of coding RNAs (17910 annotated human lncRNAs in the latest v32 version of GENCODE versus 19965 coding genes). An increasing body of evidence has highlighted characteristics that define lncRNAs as therapeutic targets as well as potential tissue-specific markers [1].

Indeed, despite their non-coding nature, a large spectrum of functional mechanisms has been associated to lncRNAs [2, 3]. These include: endogenous competition (miRNA sponging for example), protein complex scaffolding and guide for active proteins with RNA-DNA homology interactions. These mechanisms occur in various physiological or pathological processes such as development, cancer and immunity [4–6].

To date, there is no finite list of lncRNA isoforms and therefore, no complete lncRNA catalogue due to the high number of transcripts and their tissue-specific expression [7, 8]. The absence of a complete catalogue makes it difficult to establish a comprehensive lncRNA expression profile. Currently, the best strategy for the study of lncRNAs consists in the prediction of transcripts from a selection of RNAseq data in a tissue-specific condition. This strategy was successful in novel lncRNA biomarker discovery in pathological conditions [9, 10], but was poorly explored for cell lineage characterisation. Taking into account their functional importance and specificity, these RNAs should therefore not be ignored in establishing the molecular identity of a cell type.

Cell characterisation by specific markers brings different challenges such as the importance of probing the specificity of the marker and its limits in an extended number of cell types, rather than using a control/patient experimental model.

Moreover, the cells are not in a fixed state and display a variable transcriptional activity depending on cell status, environment, culture conditions and other parameters [1]. Furthermore, the lncRNAs’ function is generally poorly assessed, except in the case of recurrent known transcripts (HOTAIR, H19). The in silico elaboration of a lncRNA catalogue that document the functional domains where the candidates could act, will be beneficial in the identification of lncRNAs’ role and thus, in future experiments.

To this end, we have developed an integrated four-steps procedure consisting of: i) an ab initio transcript reconstruction from RNAseq data and characterisation of novel transcripts, ii) a differential analysis using pseudoalignment coupled with a machine learning solution in order to extract the most cell-specific candidates, iii) an original step of tissue-expression validation with specific k-mers search in large and diversified transcriptomic datasets, iv) an in-depth analysis to predict lncRNAs’ functional potential from in silico prediction approaches. The notable advantage of introducing an in silico verification using k-mers is to allow a precise and in-depth determination of lncRNAs expression profile and to quickly interrogate their lineage specificity. In addition, validation of newly identified lncRNAs has been undertaken using real-time quantitative PCR (RT-qPCR) and Oxford Nanopore Technologies (ONT) long-read sequencing.

Mesenchymal stem cells (MSCs) are defined as multipotent adult stem cells, harvested from various tissues including bone marrow (BM), umbilical cord (UC) and adipose tissue (Ad). MSCs are an interesting cell type to explore since these cells lack the extended transcriptional characterisation that could highlight their lineage belonging and/or the possibility to distinguish them from other mesodermal cell types such as fibroblasts and pericytes [11, 12]. The commonly admitted surface markers for MSCs, proposed by the International Society for Cellular Therapy (ISCT) and required to identify MSCs since 2006 are THY1 (CD90), NT5E (CD73), Endoglin (ENG, CD105) concerning the positive markers, and CD45, CD34, CD14 or CD11b, CD79alpha or CD19 and HLA-DR concerning the negative markers [13]. These markers are not distinctive and may therefore not be sufficient for the definition of cellular or biological properties. Considering their different therapeutic properties (chondro and osteo differentiation potential, immunomodulation and production of trophic factors) [14] and given the increasing usage of these cells for academic and preclinical research [15], a detailed molecular characterisation of MSCs and predictive markers of functionality will constitute an important tool in regenerative medicine. LncRNAs have emerged as a class of transcripts with tissue-specific expression and important functions, such as the regulation of MSCs function [16–18], and remain largely unexplored in these cells.

To address this need, we performed a broad transcriptomic analysis of novel lncRNAs on human MSCs. We started from publicly available MSCs RNAseq, selecting ribodepleted datasets in order to enhance lncRNAs discovery and to explore the polyA+ and polyA- lncRNAs. We restricted the differential expression analysis to a BM-MSC source compared to “non-MSC” counterpart. Once achieved, in depth in silico analysis was performed to check the lncRNAs cell specific profiles with more and extensive datasets. To validate our approach, RNAseq data from eight publicly available libraries of normal MSCs containing a large diversity of non cancerous cell types were used for novel lncRNAs detection and tissue expression comparison. We initially reconstructed more than 70000 unannotated lncRNAs present in human BM-MSCs. These lncRNAs were assigned, depending on their position relative to annotated genes, to “MSC-related long intergenic non-coding RNAs” named “Mlinc”, and to “MSC-related long overlapping antisense RNAs” called “Mloanc”. Among them, 35 Mlincs were specifically enriched in the cell lineage compared to the “non-MSC” group. Finally, after a further selection of the three most specific Mlincs, detailed in vitro and in silico functional explorations were performed.

## Results

**Fig. 1:**
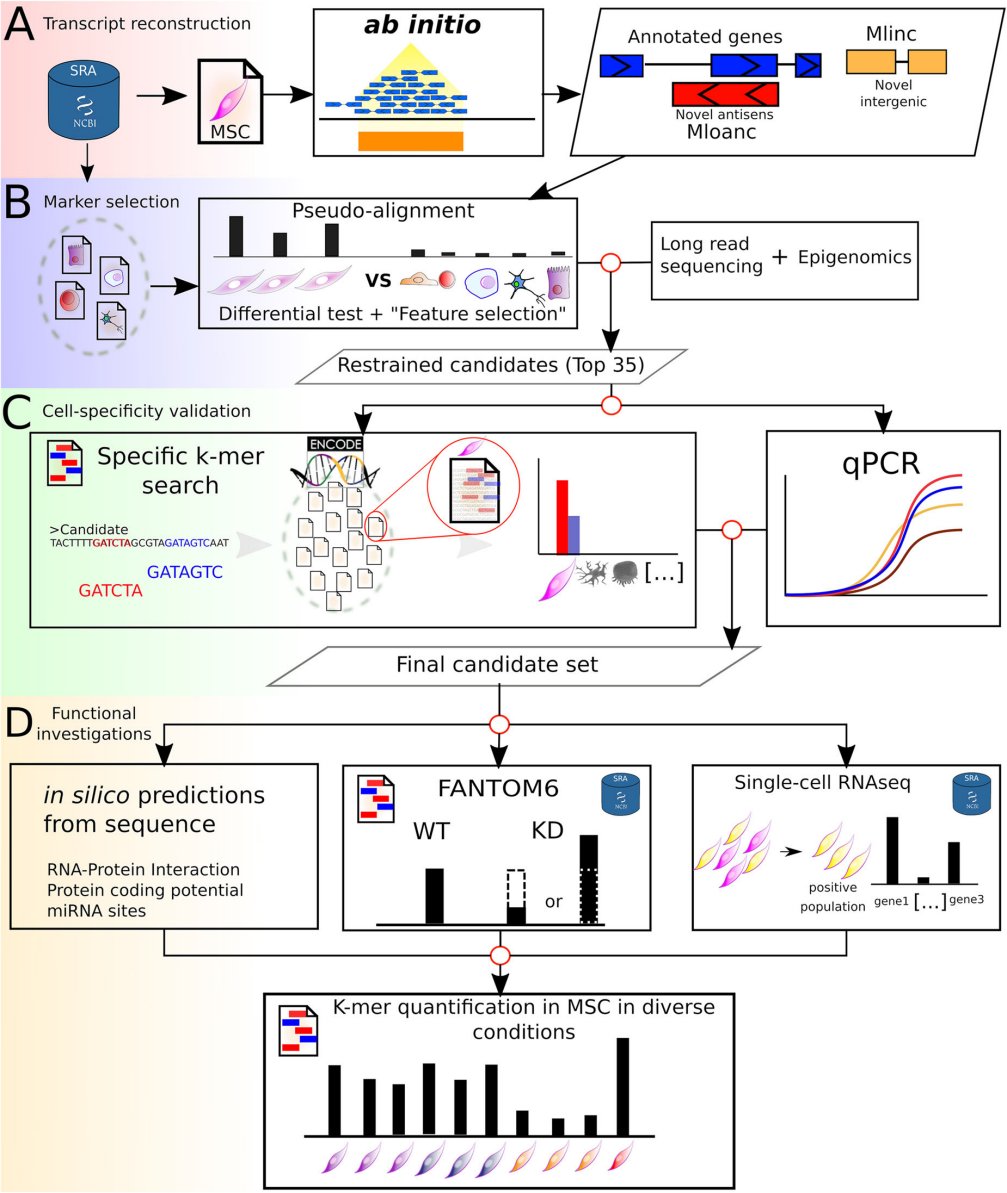
Flowchart representation of the pipeline used in this study. The 4 steps of the flowchart are described. **a** Ab initio reconstruction of transcript expressed in MSCs from SRA dataset and creation of a reference (GTF+fasta) for quantification of Ensembl annotated genes, unannotated intergenic (Mlincs) and unannotated overlapping antisens (Mloanc). The results are shown in Fig. 2. **b** Differential Analysis for the selection of MSC markers (restrained candidates set) with Kallisto pseudoalignement and Sleuth differential test followed by feature selection by random forest with Boruta package. Long-read sequencing and active transcription in MSCs by epigenetic marks information completed the selection step (see Figs. 2 and 3). **c** Validation of cell expression specificity of the candidates by k-mer quantification in ENCODE RNAseq datasets (see Additional file 8 for the list of data) and qPCR validation. The results are presented in Fig. 4. **d** Functional investigations were performed with in silico prediction methods from the sequence of candidates, followed by k-mer quantification with FANTOM6 dataset, single-cell RNAseq and selected MSC conditions. K-mer quantification phases are shown by corresponding icons (Figs. 5 and 6)

For the purpose of generating a catalogue of all transcripts in any particular cell type, we developed a pipeline for the characterisation of all RNAs and their expression profile in a large collection of RNAseq data. The procedure includes four steps: i) an ab initio transcripts reconstruction from RNAseq data and identification of unannotated transcripts, ii) a differential analysis using pseudoalignment coupled with a machine learning solution in order to extract the most cell-specific candidates, iii) an original step of tissue-expression validation with a k-mer approach (comparing large transcriptomic datasets), iv) an in-depth analysis to predict lncRNAs functional potential from in silico prediction approaches (Fig. 1). To illustrate the procedure, we produced a RNA catalogue from BM-MSCs (“MSC” group).

### General features of the predicted MSC catalogue of lncRNAs

As mentioned above, we started with the ab initio reconstruction of any transcript from BM RNAseq with Stringtie assembler after mapping the reads with the CRAC software (see “Methods” section for parameters). New isoforms of annotated transcripts were ignored. Of the 200243 transcripts present in Ensembl annotation (version 90), 105511 (52.6%) were detected in MSCs (Transcripts Per Million (TPM) >0.1 in pseudoalignment quantification).

**Fig. 2:**
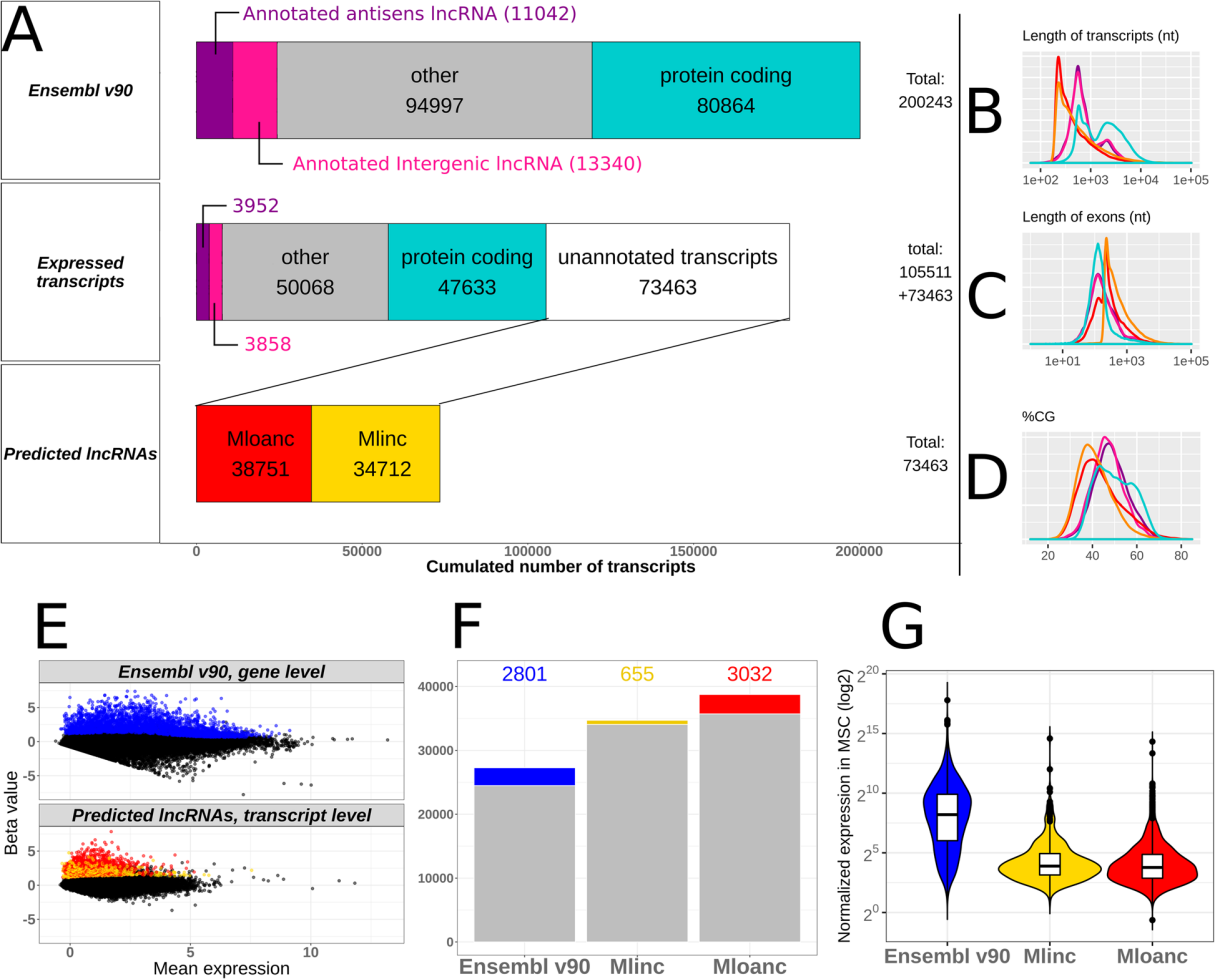
Overview of annotated genes and unannotated transcripts enriched in BM-MSCs. **a** Left pannels represented: i/ Ensembl v90 transcript categories and distribution, ii/ transcripts distribution expressed in MSCs, showing unnatotated transcripts obtained with ab initio reconstruction by StringTie vs annotated transcripts (expression >0.1 TPM), iii/ predicted lncRNAs from unnanotated reconstructed transcripts include new lncRNAs with intergenic (Mlinc) and antisens (Mlncoa) RNA categories. **b**-**c**-**d** Distribution of transcript length, exon length and GC percentage across different categories respectively with the same colors as in **a** pannel: coding transcripts (blue), annotated lincRNA (pink), annotated overlapping antisens lncRNA (purple), novel lincRNA (Mlincs, yellow), novel overlapping antisens RNA (Mloanc, red). **e** Representation of annotated genes (top pannel) and unannotated transcripts (bottom pannel) overexpresed in MSC versus non-MSC types (log2FC >0.5 and padj <0.05), separately showed in MA plot. **f** Total number of transcripts by category. The colored bar indicated the number of differentially expressed annotated genes (Ensembl v90) and unannotated transcripts (Mlinc and Mloanc). **g**Global expression in BM-MSCs (with Sleuth normalisation) of the same categories as in **f** for annotated genes and unannotated transcripts

73463 new lncRNAs were reconstructed. This fraction of unannotated transcripts represents 41% of detected transcripts, so in our case, the ab initio reconstruction made it possible to almost double the inventory of detectable signatures in MSCs (Fig. 2a). Of these, 34712 were found to be intergenic and were thus referred to as “Mlinc” RNAs, and 38751 were found to overlap with coding regions but in anti-sense orientation and thus referred to as “Mloanc” RNAs (with criteria described as in “Methods” section and Fig. 2a).

The ab initio method by itself is not sufficient to efficiently determine the lncRNAs’ full length sequences. Moreover, this step does not preclude the possibility of false positives and at this point of the analysis, all the different rebuilt transcripts are considered to be windows of RNA expression or possible artefacts. These candidates are filtered and, for the most interesting candidates, their true form is to be refined through experimental methods. We also assessed the general characteristics of predicted de novo lncRNAs in MSCs. Globally, Mlincs and Mloancs are shorter transcripts with longer exons compared to coding genes and annotated lncRNAs. The large majority of predicted lncRNAs are mono exonic (99% for Mlincs, 79% for Mloancs), with a length close to 200nt (Fig. 2b-c). A consequence of the abundance of mono-exonic lncRNAs is an infinitesimally small number of variant forms. Only 0.15% and 0.82% of Mlincs and Mloancs respectively, are not mono-isoforms. The GC content of reconstructed lncRNAs is lower than that of coding or non-coding annotated genes (Fig. 2d). This low GC proportion of around 40% is a common feature in ab initio transcript prediction, observed in a majority of studies of different species, from mammals, insects, plants or prokaryotes [19–22].

### Enrichment of a restricted set of Mlincs and Mloancs

In this second step, our objective was to obtain a restricted set of potential transcripts, using successive filtering approaches that would reveal their cell specificity. We quantified annotated transcripts, Mlincs and Mloancs with Kallisto pseudoalignment [23] in a cohort constituted of two groups: the “MSC” group containing the BM-MSCs initially used for ab intio reconstruction and the “non-MSC” group, used for comparison, composed of a large panel of different cell types including human embryonic stem cells (hESC), hematopoietic precursors and stem cells, primary chondrocytes, induced pluripotent stem cells (iPSCs), hepatocytes, neurons, lymphocytes and macrophages (metadata available in Additional file 1).

Only over-expressed transcripts in “MSC” group versus “non-MSC” group were selected. Differential statistical tests were made with Sleuth, a tool specially dedicated to Kallisto quantification results [24] (see all selective parameters in “Methods” section). We performed two differential expression analyses: one at the gene level for Ensembl annotation and the other at the transcript level for unannotated transcripts, to give the most likely variant form of the predicted lncRNAs. After this differential analysis, 2801 annotated genes, 655 Mlincs and 3032 Mloancs are significantly overexpressed in BM-MSCs (Fig. 2e-f).

The lncRNAs are commonly known to be less expressed than coding genes and this was observed in our selected annotated genes and new lncRNAs (Fig. 2g). As a validation of our procedure, we found the 3 positive MSC markers of ISCT among the selected annotated genes: THY1 (CD90), ENG (CD105), and NT5E (CD73). We also retrieved some influencers of MSCs activity, for example WNT5A [25, 26], Lamin A/C [27] and FAP [28]. The complete list of selected genes is provided in Additional file 2.

### Feature selection for the most discriminating coding and non-coding markers

In an attempt to select the best candidates, we retained lncRNAs with the most discriminating profile between “MSC” and “non-MSC” groups. In our case, the limitation with a classical “top” ranking by fold change (FC) or *p*-value is the presence of subgroups of different cell types inside the “non-MSC” group. The FC, estimated by the Beta value in Fig. 2c, appears to be a biased indicator of differential expression as it can select strong but localised expressed lncRNAs in cells poorly represented in our control group, leading to potential false positive results.

**Fig. 3:**
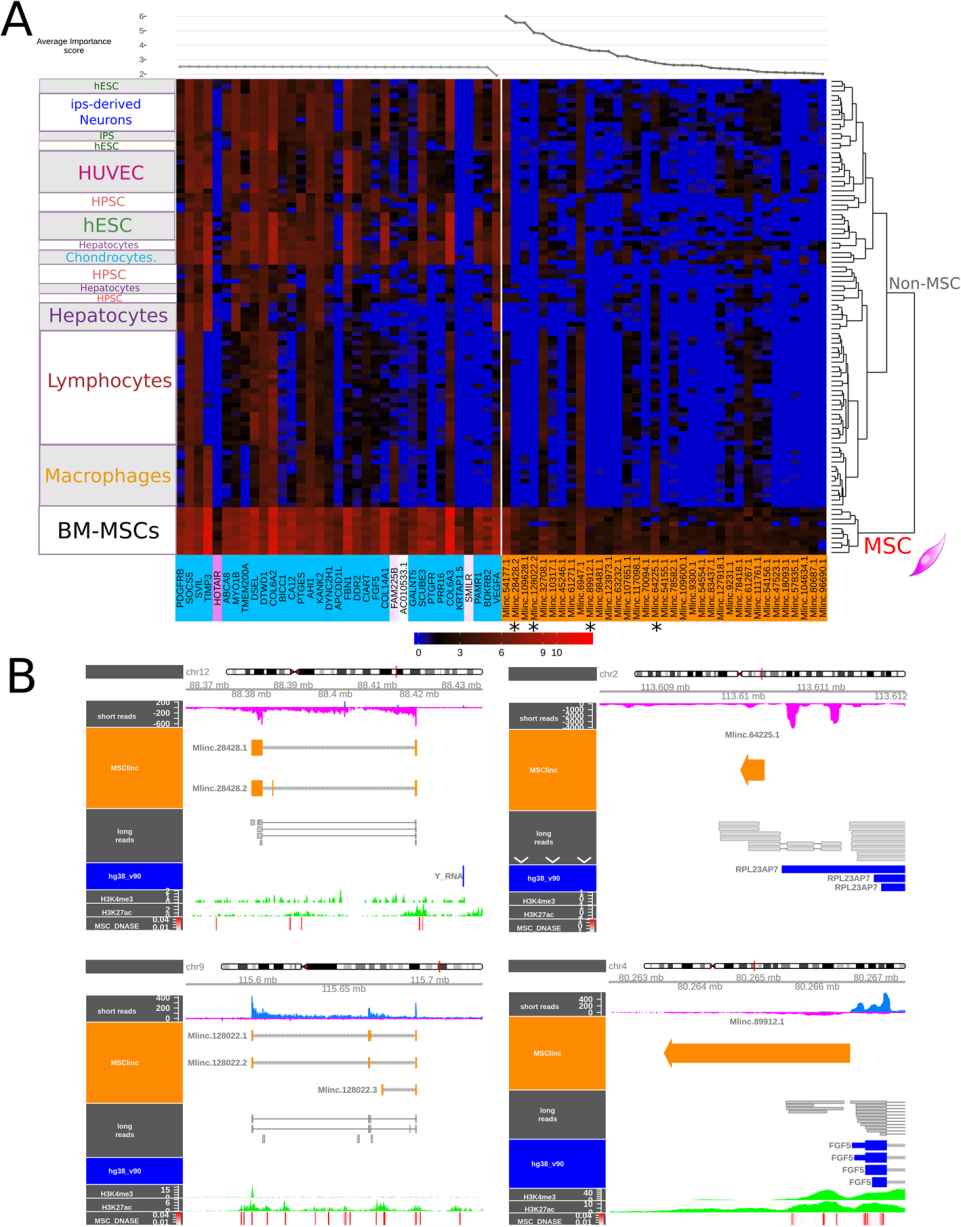
Selection of a refined set of the best candidates by random forest (top35), long-read sequencing and epigenetic features. **a** Expression of the best MSC-specific candidates selected by Boruta machine learning along MSC group and not MSC cohorts. Left pannel: top35 most relevant annotated genes (non-coding included); Right pannel: unannotated intergenic lncRNAs (Mlincs) and their average importance scores determined by Boruta method displayed in upside line plot. **b** Genomic visualisation of Mlincs 28428 (up left panel), 64225 (up right panel), 128022 (down left panel), and 89912 (down right panel). Predictions (Mlinc orange) from short reads alignment of all MSC group files (blue/magenta and BAM visualisation), are compared with unoriented long-read alignments (grey). Additional epigenomic features are shown to reveal active transcriptional activity from trimethylation of Histone H3 (H3K4me3), acetylation of Histone H3 H3K27 in MSCs (H3K4me3 and H3K27ac, green), and Dnase sensibitity hotspots of MSC (MSC DNAse, red)

To avoid this problem, we used the Boruta feature selection [29] (see “Methods” section), to select discriminating features based on random forest machine learning methodology. Boruta was used separately on each group of candidates (annotated genes, Mlincs and Mloancs) to extract a restricted representation of the most relevant MSC signatures. The top 35 importance scores were selected for annotated genes, Mlincs and Mloancs. We arbitrary chose to select the first 35 transcripts for each group based on the observation of the importance score. Considering the expression profile of these top 35 coding genes and predicted Mlincs, BM-MSCs clusterised independently from other cell types (Fig. 3a). In contrast, the selection of Mloancs did not provide a satisfying clustering as they had similar expression profiles in MSCs and other closely related cell types, in particular in primary chondrocytes (Figure in Additional file 3). For this reason, Mloancs were not retained for further analysis. Selected annotated genes showed a poor specificity, with only few candidates showing a clear difference of expression between MSCs and others: APCDD1L, HOTAIR, KRTAP1-5 and SMILR. The 3 positive MSC markers from the ISCT were absent in this selection. The novel top 35 Mlincs showed less expression overall but with a more distinctive profile and a higher number of possible MSC markers with clear contrast of expression. The characteristics, genomic intervals and sequences of the 35 candidates are presented in Additional file 4.

To assess the potential of genes already proposed as potential MSC biomarkers by ISCT (Figure in Additional file 5) or other potential MSC markers proposed by different authors [14] (Additional file 6), we made a separated expression heatmap without filter. Among these previously proposed markers, THY1 (CD90) presented the most specific profile. However, each gene is expressed in distinct non-MSC types.

### Validation of selected Mlincs with long-read sequencing

As mentioned above, classical annotation of lncRNAs with ab initio short read methods suffers from inaccuracies and biases. The ONT can sequence entire cDNA, which constitutes a clear technological advantage, not only in confirming the existence of the transcripts but also as it makes it possible to precisely identify the genomic intervals of lncRNA candidates. We performed long-read sequencing of a polyA+ RNA library obtained from a BM-MSC sample. Among the top 35 selected Mlincs, 4 transcripts are covered with the ONT sequencing, in 3 million total reads.

These intergenic lncRNAs are named as Stringtie output (“SetName. TranscriptNumber. VariantNumber”): Mlinc.28428.2, MlincV4.128022.2, MlincV4.89912.1 and MlincV4.64225.1. To support the above transcriptional units, we compared them with our short read data and searched for epigenetic status at the locus of the Mlincs in BM stromal mesenchymal cells. We looked at DNase sensitive site, H3K27 acetylation, H3K4 trimethylation that commonly corresponds to active regulatory regions (Fig. 3b) and 5’ Cap analysis of gene expression (CAGE) experiments of ENCODE/RIKEN (Additional file 7), collected from UCSC genome browser (see “Methods” section).

We globally observed a DNA accessibility enrichment and acetylation of Histone 3 at the promoter region of our candidates, correlating with DNAse sensitivity hotspots in BM mesenchymal cells that reinforce the prediction of the expression windows. In particular, for Mlinc.28428.2, the transcript observed with long-reads sequencing corresponded to the prediction made with short reads. It was also supported by Mlinc.28428.1, a variant that differs by the absence of the second exon. Similar characteristics were observed for Mlinc.128022, which also produced two variants with a different organisation of 5 exons. The two other candidates, Mlinc.89912.1 and Mlinc.64225.1, are mono-exonic. Mlinc.89912.1 occurs at the close proximity of FGF5 3’end, in reverse orientation. For this reason, the different epigenomic features could not be attributed with certainty to the Mlinc. For Mlinc.64225.1, the long-read sequence is longer than the ab initio short read prediction. Except for Mlinc.64225, and in accordance to the start of long reads, we observed CAGE enrichments at the 5’ end of Mlinc predictions in MSCs polyA+ libraries. This CAGE enrichment was not observed for CD34 cells and hESCs polyA+ libraries. This observation validates both the intervals and the existence of a polyA form of these candidates (Additional file 7). KRTAP1-5, HOTAIR and SMILR, selected for their good expression profiles, were also covered by long reads (Data not shown).

### High-throughput investigation of a marker’s specificity by specific k-mers search

A marker can only be considered specific within the limits of the diversity of samples used for its study. Considering the growing number of cells/tissues and transcriptional profiles, it is essential to probe the limits of a chosen biomarker against these various cell types. Most of published analyses highlighting new potential biomarkers of MSCs or fibroblasts have been restricted to a comparison between only few cell types and, as discussed, commonly described markers are not strictly distinctive. In order to assess the expression of Mlinc candidates in a large number of samples, we extracted specific 31nt k-mers from each of their sequences, as previously described [30]. These simplified but candidate-specific (oligonucleotide-like) probes allow a simple and fast presence/absence search on large-scale cohorts and a direct quantification in raw FASTQ data. The k-mers were quantified in ENCODE human RNAseq database, including “primary cells” and “in vitro differentiated cells” categories (Additional file 8). Particularly, as the bibliography suggests that MSCs can also express phenotypic characteristics of endothelial, neural, smooth muscle cells (SMCs), skeletal myoblasts and cardiac myocytes, RNAseq samples from this mesodermal origin were tested.

**Fig. 4:**
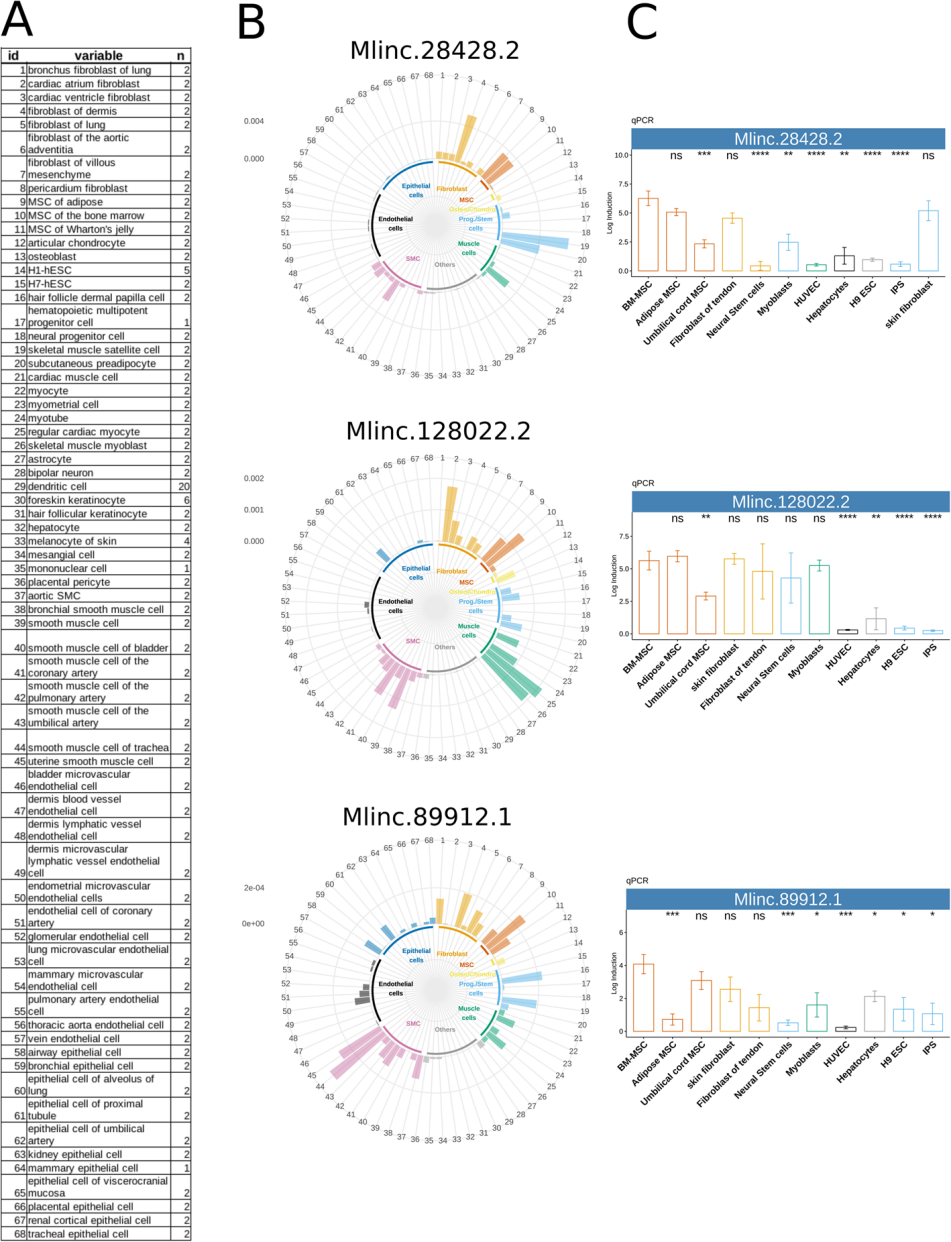
High throughput exploration of selected candidates across a variety of samples by k-mer quantification in RNAseq and biological validation by RT-qPCR. **a** List of tissues for the cell specific expression exploration (samples with ID numbers are listed in Additional file 8) **b** Relative expression of Mlinc.28428.2, Mlinc.128022.2, and Mlinc.89912.1 across ENCODE’s ribodepleted RNAseq data, made by k-mer quantification, normalised by k-mer per million. **c** qPCR relative quantification was performed on the selected 3 Mlincs in MSC of different origins (BM-MSC, Ad-MSC, Umbilical cord msc) and other indicated cell types. Relative quantification (Log induction) was quantified by ddCt method using non MSC types as calibrator (mean of triplicates). Student tests have been made between triplicates, each test using BM-MSCs as reference group (ns: P >0.05, *: P ≤0.05, **: P ≤0.01, ***: P ≤0.001, ****: P ≤0.0001)

With ISCT positive markers, we observed an expected expression profile that recapitulates previous biological studies, particularly the high expression of ENG (CD105) in endothelial cells (Figure in Additional file 9) and the overexpression of NT5E (CD73) in epithelial and endothelial cells (Figure in Additional file 10). Interestingly, their expression varied among MSC sources: NT5E (CD73) was strongly enriched in Ad and BM derived MSCs and THY1 (CD90) in UC derived MSCs (Figure in Additional file 11). We next analysed the expression profile using our candidate annotated genes Mlinc specific k-mers (Fig. 4). The specific k-mers search supported the stated expression profile of Mlincs previously shown: our Mlinc candidates were positive in MSCs and displayed low or absent expression in cells of ectodermal lineage, hematopoietic or endothelial origins.

However, the high throughput and naive quantification in the ENCODE cohort made it possible to extend the observation of this absence of expression into cell types not previously studied. Moreover, this diversity showed that the expression of most of the candidates, contrary to positive markers of the ISCT, were exclusive of cells with mesodermal origin. All candidates were expressed in at least one type of fibroblasts and differentially present in other mesodermal cell types. For the 4 selected Mlincs, they shared (i) a systematic and strong expression in cell types like skin fibroblasts and cells derived from reservoir of mesenchymal progenitors (muscle satellite cells or dermis papilla cells), (ii) a homogenous over-expression in regular cardiac myocytes, and (iii) an irregular expression in SMCs. The ENCODE cohort containing MSCs of different origins, we can therefore observe that the Mlincs show differences of expression depending of the tissular origin, these candidates being mainly expressed in two MSC types. The results permitted the classification of our Mlincs according to observed specificity, from the most promising to the least restricted profile: Mlinc.28428.2 is expressed in Ad and BM derived MSCs. It is the candidate with the clearest absence of expression in non-mesodermal cells and with the poorest relative expression in SMCs. Mlinc.128022.2 is expressed in Ad and BMMSCs and particularly in preadipocytes and muscle cells (myoblasts, myocytes and myotubes). Mlinc.89912.1 is principally expressed in BM-MSCs and less in UC and Ad-MSCs, but shows expression in epithelial and endothelial cells. Finally, Mlinc.64225.1 differs from other Mlincs as it is also strongly expressed in keratinocytes, hematopoietic stem cells and epithelial cells (Figure in Additional file 12). Its expression in non-MSC types, has led us to retain the 3 other Mlincs for further investigations.

### RT-qPCR mimics the in silico prediction and deciphers multiple transcript variants

To confirm the specificity of selected Mlincs’ expression experimentally, we performed RT-qPCR on a set of 80 RNA preparations from different primary cells (Fig. 4c). These include MSCs from BM, Ad and UC, fibroblasts of different tissue origins, iPSCs, neural stem cells, myoblasts, human umbilical vein endothelial cells (HUVECs) and hepatocytes. RT-qPCR and amplicon sequencing using sets of specific primers (Additional file 4) confirmed different predicted forms of the Mlinc candidates in BM-MSCs (Additional file 13). We designed two primer pairs for both Mlinc.128022 variants to validate the existence of first splice, and two pairs for Mlinc.28428 variants, one overlapping the second exon and another corresponding to a splice between first and third exons. All variations captured by the primers design were quantified, suggesting that all these different variations predicted in silico exist biologically in MSCs. We confirmed most of the expression profiles obtained by k-mers quantification using RT-qPCR, notably the specificity of expression dependency on the MSC tissular origin: over expression of Mlinc.28428 and 128022 in BM and Ad-MSCs. Nevertheless, few exceptions such as Mlinc.89912.1, presented an enrichment in UC-MSCs not found with k-mers quantification. Moreover, the restricted expression to cells of mesodermal origin is confirmed in our RT-qPCR results. We obtained similar observations with annotated candidates: overexpression of KRTAP1-5 and SMILR in BM-MSCs specifically, and of HOTAIR in UC and BM-MSCs.

### In silico prediction of lncRNA interactions and functions

The relative specificity of selected Mlincs for mesenchymal cells could be an indication of their roles in MSCs function. The prediction of their possible function could therefore suggest their suitability as markers of MSCs’ function potential. To this end, we explored assumptions on the function of Mlinc.28428.2, Mlinc.128022.2 and Mlinc.89912.1 candidates using different published methods. We first used bioinformatic tools based on machine learning and deep learning to decipher general characteristics of our candidates: FEELnc [31] to assess coding potential, tarpMir [32] to decipher “miRNA sponge” function and LncADeep [33] to analyse potential interactions with proteins. Only two of the 35 selected Mlincs and none of the 3 selected Mlincs with validated specificity were revealed as potentially coding RNAs, the majority being predicted as non-coding by FEELnc (33/35). None candidate had more than five target sites for a given miRNA, indicating a low probability of a “miRNA sponge” activity (Additional file 4). For the 3 retained Mlincs, predicted interacting proteins by LncADeep were submitted to Reactome (Additional file 14). We noted a predicted interaction between Mlinc.28428.2 and Betacatenin (CTNNB1) as part of apoptosis-linked modules, 5’-3’ Exoribonuclease 1, component of the CCR4-NOT complex, mRNA Decapping Enzyme 1B as part of the mRNA decapping and decay pathways. The interaction was also predicted with different mediators of RNA polymerase II transcription subunits (MED), ATP Binding Cassette Subfamily B Members as part of the PPARA activity linked to ER-stress [34], and Proteasome subunits for intracellular transport, response to hypoxia and cell cycle modules. Mlinc.128022 could interact with important genes like THY1 (CD90), NRF1 (mitochondria metabolism) with no module clearly highlighted. Mlinc.89912 could interact with tubulins, UBB (ubiquitin B), SMG6 nonsense mediated mRNA decay factor and ribosomes subunits (RPSX) proteins, RPL24 for nonsense mediated decay (NMD), PINK1 (mitophagy) and finally MGMT as part of the MGMT mediated DNA damage reversal module.

**Fig. 5:**
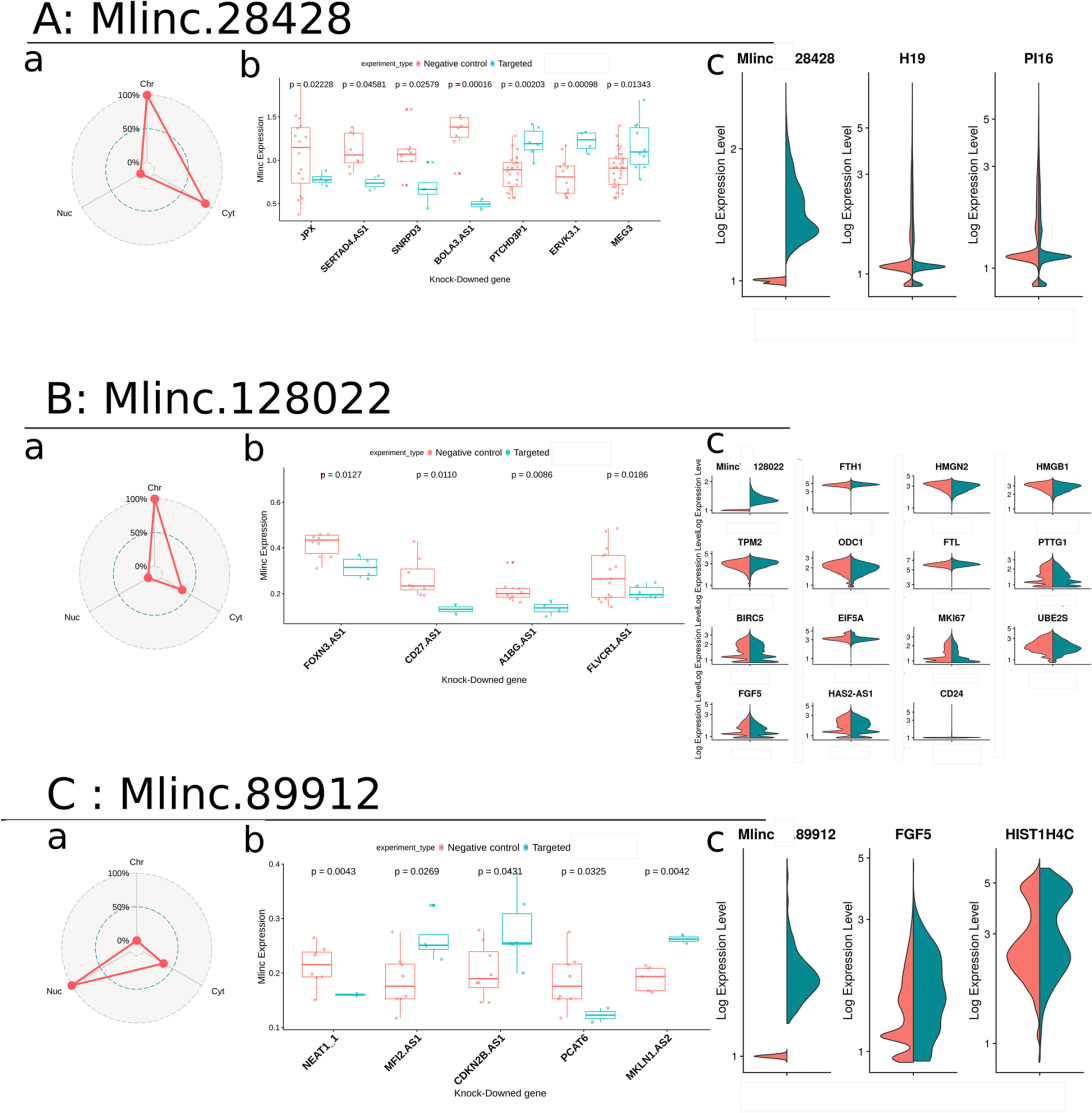
Prediction of potential functions of the candidates with k-mer quantification and single-cell. For each Mlinc (Mlinc.28428 (**a**) Mlinc.128022 (**b**) and Mlinc.89912 (**c**) respectively) 3 steps of prediction were performed. **a** Enrichment in the different subcompartments of fibroblasts from FANTOM6 dataset: free nuclear fraction (Nuc), chromatin (Chr) and cytoplasm (Cyt); **b** Expression of markers in FANTOM6 data depending of the Knock-down (KD) of annotated lncRNAs. Normalised counts of all specific k-mers is averaged by sample (zero values deleted) and t-tests are made between control (in pink) and KD fibroblasts (in turquoise). **c** General expression of Mlincs inside Ad-MSC population, dimensional reduction made with UMAP method, made from batch corrected counts. Expression of differentially expressed annotated genes between positive (in turquoise) and negative (in pink) cells for Mlinc.28428, Mlinc.128022 and Mlinc.89912 respectively

We further quantified the expression of our candidates by counting their specific k-mers in the entire FANTOM6 set of 154 Knock-downed (KD) annotated lncRNAs in human dermal fibroblasts (10.1101/700864, dataset presented in Additional file 15). We selected the KD experiments where expression of the Mlincs was statistically differential when compared with controls. Particular attention was paid to KD lncRNAs with reported function(s) in bibliography and to KD lncRNAs overlapping a gene with reported functions. Mlinc.28428.2 is down-regulated when JPX, SERTAD4-AS1, BOLA3-AS1, and SNRPD3 are KD and over-expressed with the KD of PTCHD3P1, ERVK3.1 and MEG3, among other lncRNAs without reported function (Fig. 5a). Interestingly, interactions between p53 pathway and JPX [35], SNRPD3 [36] and MEG3 [37, 38]) respectively, have been previously reported. All these features converge on the hypothesis of a link between the function of Mlinc.28428, stress response, senescence and cellular maintenance. The implications of BOLA3 [39, 40]) and PTCHD3P1 [41] in mitochondria homeostasis and glycolysis, the role of BOLA3 in stress response [42], the status of SERTAD4 as a target of the YAP/TAZ pathway [43], vital pathway of stress response [44], and the role of MEG3 in aging [45], all reinforce this hypothesis.

Mlinc.128022.2 is down-regulated with the KD of FOXN3-AS1, A1BG-AS1, CD27-AS1, and FLVCR1-AS1 (Fig. 5b). FOXN3 seems to be more than a regulator of cell cycle, it is also described as a regulator of osteogenesis in different cases of defective craniofacial development [46, 47]. Moreover, the reported over-expression of FOXN3 during the early stages of MSC osteodifferentiation [48], and the down-regulation of CD27-AS1 in MSCs of donors with bone fracture [49], allow us to hypothesise a possible function of Mlinc.128022 in bone remodelling and osteogenesis. In addition, both A1BG-AS1 and FLVCR1-AS have an influence in osteogenesis and cell differentiation. A recent study showed that A1BG-AS1 interacts with miR-216a and SMAD7 in suppressing hepatocellular carcinoma proliferation [50], both partners having an important role in the positive regulation of osteoblastic differentiation in mice [51, 52]. FLVR1 participates to the resistance of oxydative stress by heme exportation in mouse MSCs [53], iron metabolism being closely linked with bone homeostasis, formation [54] and cell differentiation [55].

Finally, Mlinc.89912.1 is down-regulated after the KD of NEAT1-1 and PCAT6, and over-expressed when MFI2.AS1, CDKN2B.AS1 (or ANRIL) and MKLN1.AS2 are KD (Fig. 5c). The manifest relations between cell proliferation and CDKN2B-AS1 [56, 57], MFI2 [58], MFI.AS1 [59], PCAT6 [60] and NEAT1 [61, 62], a combination with the DNA damage repair response, [63, 64] reinforce the prediction of a role of Mlinc.89912 in these mechanisms. Moreover, we explored RNAseq from chromatin, nucleus and cytoplasm subcellular compartments of fibroblastic cells in the FANTOM6 Dataset. Mlinc.28428 and Mlinc.128022 are enriched in at least cytoplasm (Fig. 5a-b), whereas Mlinc.89912 is enriched in free nucleus fraction suggesting interactions with nuclear component (Fig. 5c).

### The single-cell RNAseq: an emergent level of completion in marker search

We analysed the single-cell RNAseq (scRNAseq) data from 26071 Ad-MSCs to assess the heterogeneity of the 3 Mlincs, to explore their expression at the single-cell level (dataset presented in Additional file 16) and to provide a supplemental layer of functional investigation. No clear correlation between cell cycle and expression of our Mlincs was identified (Additional file 17). We observed a high variability of the number of cells expressing the markers (Threshold ≥0.1). 11927/26071 were Mlinc.28428-positives, 4944 were Mlinc.128022-positives, and 404 were Mlinc.89912-positives. For each Mlinc, we performed a differential test to decipher genes differentially expressed in Ad-MSCs Mlinc-positive and Mlinc-negative cells.

We found that Mlinc.28428-positive cells under-express H19 and PI16 (Fig. 5a). These genes, that present a diversity of functions, are involved in stress mechanisms (oxydative response and shear stress), inflammation in fibroblasts and MSCs and senescence pathways [65–68]. Despite the low number of differentially expressed genes in Mlinc.28428-positive cells, their functional behaviour and their known targets suggest a pathway linked to stress response and senescence establishment that reinforce our previous assumptions on Mlinc.28428 function.

Mlinc.128022-positive cells are enriched in FTH1, TPM2, FTL and CD24 and present a lower expression in HMGN2, HMGB1, ODC1, PTTG1, BIRC5, EIF5A, MKI67, UBE2S, FGF5, HAS2-AS1 (Fig. 5b). A significant portion of these genes are linked to osteogenic properties of MSCs as previously observed with FANTOM analysis. The Mlinc.128022-positive cells have an increased expression of ferritin (light and heavy chains), major actor in iron metabolism in osteoblastic cell line [69], that is also involved in osteogenic differentiation [70] and osteogenic calcification [71]. Two genes, enriched in Mlinc.128022-positive cells, are positively linked to the osteogenic differentiation potential of MSCs: the tropomyosin 2 (TPM2), downregulated when human MSCs were cultured in OS medium for the induction of osteoblasts at the calcification phase [72], and CD24 a membrane antigen recently proposed as a new marker for the sub-fraction of notochordal cells with increased differentiation capabilities [73]. In addition ODC1, under-represented in Mlinc.128022-positive cells, inhibited the MSCs osteogenic differentiation [74, 75]. Finally, the decrease of FGF5, MKI67, BIRC5 (survivin) and PTTG1 (securin) expressions, all linked to proliferation active phases of cell cycle, tend to show cell with arrested cell cycle. These data suggest that the expression profile of Mlinc.128022 positive cells indicate a subpopulation of undifferentiated osteogenic progenitors, probably in senescence or quiescence.

Mlinc.89912-positive cells are enriched in FGF5 and HIST1H4C (Fig. 5c). FGF5 is a protein with mitogenic properties, identified as an oncogene, that facilitates cell proliferation in both autocrine [76] and paracrine manner [77]. HIST1H4C, the Histone Core number 4, is a cell cycle-related gene. Modification of histone H4 (post-transcriptional or mutation) has been highlighted as important for non-homologous end-joining (NHEJ) in yeast [78]. Its mutation causes genomic instability, resulting in increased apoptosis and cell cycle progression anomalies in zebrafish development. It reinforces our assumptions that Mlinc.89912 has a role in cell proliferation and DNA damage repair. In conclusion, the scRNAseq analysis enabled the observation of different features that characterise the phenotype of Mlincs positive cells and reinforced hypotheses on their functions previously observed through k-mers quantification.

### K-mers analysis of markers in functional cell situation

Previously, we have presented a number of strategies to formulate hypotheses on the functions of unannotated lncRNAs, suggesting directions of future experimental investigations. To evaluate the relevance of these strategies, we sought to quantify with specific k-mers search the expression of our Mlincs in MSCs in different conditions, linked to above mentioned findings: stress and senescence for Mlinc.28428.2, osteodifferentiation for Mlinc.128022.2 and cell cycle/proliferation for Mlinc.89912. We downloaded RNAseq data corresponding to the above-mentioned focus, described in Additional file 18.

**Fig. 6:**
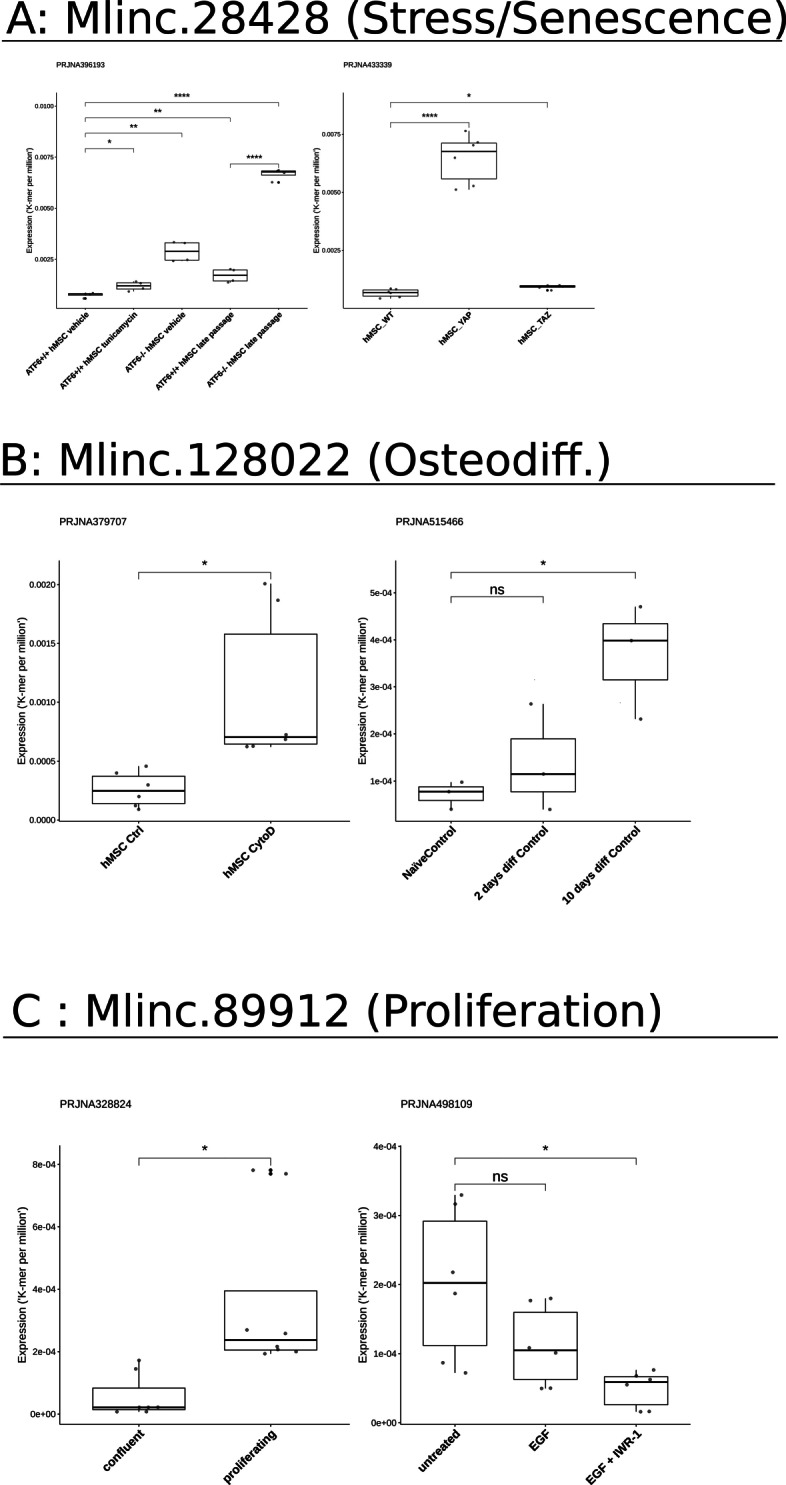
Expression of markers in different datasets from SRA in cell conditions related to previous findings. **a** Expression of Mlinc.28428.1 in the context of oxydative, replicative, or KO-driven, stress and senescence (PRJNA396193, PRJNA433339). Relevant changes of expression are showed with t-test results (ns: P >0.05, *: P ≤0.05, **: P ≤0.01, ***: P ≤0.001, ****: P ≤0.0001). **b** Expression of Mlinc.128022 in osteodifferentiation conditions (PRJNA515466) or osteodifferentiation potential (PRJNA379707). Relevant changes of expressions are showed with t-test results (ns: P >0.05, *: P ≤0.05, **: P ≤0.01, ***: P ≤0.001, ****: P ≤0.0001). **c** Expression of Mlinc.89912 in the context of proliferation (PRJNA328824 and PRJNA498109). Relevant changes of expression are showed with t-test results (ns: P >0.05, *: P ≤0.05s, **: P ≤0.01, ***: P ≤0.001, ****: P ≤0.0001). The detailed list of datasets is provided in Additional file 16

As shown in Fig. 6, we observed a statistically relevant increase of Mlinc.28428 expression in MSCs under replicative stress and in MSCs with CRISPR-Cas9 depletion of genes with important role against senescence. In the Wang et al. study [79], MSCs senescence was observed with the knockout (KO) of ATF6 and the stress induced with tunicamycin (endoplasmic reticulum stress) and late passage (replicative stress). Mlinc.28428 expression increased with tunicamycin treatment, late passage and ATF6 KO. The highest increase is observed in ATF6 KO MSCs associated with late passage condition.

In Fu et al. study [80] YAP, but not TAZ, was found to safeguard MSCs from cellular senescence as shown by KO experiments. Interestingly, YAP KO, significantly increases the expression of Mlinc.28428.2. This would lead us to conclude that Mlinc.28428 is overexpressed in senescence and stress conditions, suggesting a role in one or both of these phenomena.

The change in Mlinc.128022 expression is strictly linked to osteodifferentiation conditions. Mlinc.128022 expression shows a relevant increase in MSCs exposed to fungal metabolite Cytochalasin D (CytoD). The CytoD is reported as an osteogenic stimulant in the concerned study [81]. Moreover, no expression variation was observed between MSCs and MSC-derived Ad from Wang et al. study, implying a role in adipodifferentiation. Agrawal Singh et al. studied osteogenic MSCs differentiation [82], with a similar increase of Mlinc.128022 being observed after ten days.

We then quantified the expression of Mlinc.89912 in a study that compares proliferating MSCs versus confluent MSCs [83, 84]. Our candidate was clearly over-expressed in proliferating cells, validating its capacity to mark the MSCs in proliferation. Moreover, its expression was not statistically modified when MSCs were exposed to epidermal growth factor with pro-mitotic capabilities [85]. However Mlinc.89912 expression was reduced when IWR-1, an inhibitor of beta-catenin nuclear translocation, that reduced the proliferation of MSCs, was added to the medium. The functional domains of these genes are summarised in Table 1 and confirm the potential functional role suggested from FANTOM data: stress-related pathways for Mlinc.28428, MSCs differentiation with a presumed orientation in osteo-progenitors for Mlinc.128022 and a more restricted role in proliferation and DNA repair for Mlinc.89912.

**Table 1:** Results of functional investigations’ summarised for each of the three selected Mlincs

Mlinc	Predicted RNA-Protein interactions (lncADeep)	Subcompartment enrichment	FANTOM6 expr. changes	Diff. genes in positive cells	K-mers investigations
Mlinc.28428	Apoptosis, mRNA decay, PPARA activity, intracellular transport, response to hypoxia and cell cycle	Chromatin, cytoplasm	BOLA3-AS1, JPX, SERTAD4-AS1, PTCHD3P1, ERVK3.1, SNRPD3, MEG3	H19, PI16	Stress, senescence
Mlinc.128022	THY1, NRF1	Chromatin, cytoplasm	FOXN3-AS1, A1BG-AS1, CD27-AS1, FLVCR1-AS1	FTH1, TPM2, FTL, CD24, HMGN2, HMGB1, ODC1, PTTG1, BIRC5, EIF5A, MKI67, UBE2S, FGF5, HAS2-AS1	Osteodiff., Stress
Mlinc.89912	MGMT-mediated DNA damage reversal, Nonsense Mediated Decay, Tubulin metabolism	nucleus (free), cytoplasm	NEAT1_1, PCAT6, MFI2-AS1, MKLN1-AS2, CDKN2B-AS1	FGF5 and HIST1H4C	Proliferation

## Discussion

With recent evolution of omics analysis, the landscape of biomarkers has been extended beyond known genes to the unexplored transcriptome. This potential has been assessed in pathological conditions but to a lesser extent in cell-specific conditions, where this new pool of potential markers could be used to identify less well-characterised cells and hence predict their function. In this article, we propose an integrated procedure and strategies to identify the best markers (annotated or not) in a cell-specific condition, and predict their potential functions, primarily from RNAseq data (Fig. 1). RNAseq facilitates the creation of large lncRNA catalogues [8, 86], however it remains incomplete given the diversity of biological entities and lncRNAs specific expression in non-pathological, cell-specific conditions. The creation of a “home-made” catalogue associated with a specific condition remains the best way to assess the full diversity of potential biomarkers in a cell, rather than resorting to a global catalogue made from diverse samples. To give an idea of the completeness of such a focused lncRNA catalogue when compared to a global one, Jiang et al. recently published “an expanded landscape of human long non-coding RNA” with 25 000 new lncRNAs from normal and tumor tissues, whereas in our focused analysis only 50% of our 35 selected Mlinc can be found in this collection [86].

Futhermore, providing new candidates of good quality to improve lncRNA collection remains a complex task. As it could be expected, the raw catalogue in our study contains predictions of disparate quality observed with a large number of mono-exonic transcripts. Without any filter, ab initio methods are insufficient to adequately reconstruct full length transcripts. The usage of long-read sequencing has been particularly effective in helping to validate our predictions. Given the benefits of full-length RNA sequences, long-read sequencing should become the standard for lncRNA validation. A specific lncRNA can be the one presenting the most relevant properties after in silico analysis. The first task remains the identification of the more specific markers for a given cell type, task that present differences from classic comparative analysis. The MSC markers proposed in the past were determined through a simple comparison between MSCs of a certain origin with non-MSC cells whose types are either unique or few in number.

Historically, MSCs have been compared to BM hematopoietic stem cells. However, our initial RNAseq analysis revealed that all potential MSC markers proposed in the past are expressed in at least one other non-mesenchymatous cell type, and so, do not constitute exclusive MSC markers at the transcriptome level. Even if all cell types cannot be investigated, the diversity of the negative cell set is a critical criterion in selecting the most specific transcripts. In keeping with this idea, we restricted the list of potential biomarkers with an enrichment step based on a differential expression comparing BM-MSCs to other cells including stem cells, as well as differentiated cells of various lineages (lymphocytes, macrophages, primary chondrocytes, hepatocytes and neurons). In the enriched list, the overexpressed annotated genes contained members of MSC-related pathways as well as the ISCT markers. This result supported the MSCs characterisation made by the original authors [13], thus validating the identity of MSCs used for this RNAseq analysis with the currently defined criteria. The problem with classical differential analysis used on diverse “non-MSC” group is that all the group is considered to be homogeneous. As a result, candidates with positive expression in small cell groups could pass statistical test, creating false positives. For this kind of differential analysis, we propose to select the most discriminating transcripts by feature selection, a machine learning methodology that reduces the number of non-discriminating candidates after selection. We used feature selection through Boruta, a method based on “random forest”, to retain the top 35 most relevant MSCs signature for annotated genes, Mlincs and Mloancs separately. Putting aside our initial focus on unannotated lncRNAs, different annotated lncRNAs or coding genes with interesting profiles were also selected by feature selection: among them, KRTAP1-5 have been exclusively studied in BM-MSCs [87], where its preferential expression was validated by our results. These discoveries can bring new features concerning these genes and suggest directions for future investigations concerning their impact on the MSCs.

However, a marker is classically considered as specific on condition that its positive expression cannot be observed in any other cell type. Therefore, the expression of these potential markers should be explored in an entire RNAseq database to further validate its specificity. The exploration of a wide set of RNAseq data as proposed by ENCODE, including a diversified set of primary and stem cells, could support or invalidate the specificity of potential markers. In order to assess the expression of Mlinc candidates in a large number of samples, we used a signature for each candidate, extracting specific 31nt k-mers from their sequences. The specific k-mers extraction was made using Kmerator software. These k-mers were then quantified in the ENCODE human RNAseq database. The new and simplified procedure based on k-mers counting and large scale RNAseq exploration has the following advantages: i) a direct textual search that requires less time and CPU resources than classical methods and ii) a restricted set of lncRNAs supported by different results in the biological (wet) and in silico levels (RNAseq data). The counterpart of the extensive vision of marker expression is that we observe a limit of specificity among our best candidates. We observed expression in fibroblasts, in close primary cells of common embryonic origin like SMCs and other tissue-specific fibroblastic cells. Other tissue resident fibroblastic cells like skeletal muscle satellite cells, pre-adipocytes and fibroblasts from different sources, especially dermis, express our selected Mlincs markers. The question of the differences between MSCs and related cell types is crucial to the issue. Specifically, the differences between MSCs and fibroblasts remain a subject of debate [12, 88]. According to the ISCT statement, no phenotypical differences have been reported between fibroblasts of different sources and adult MSCs [89], suggesting the hypothesis of a uniform cell type with functional variation depending on the tissue source. Our results support this idea: distinguishing MSCs from fibroblasts with only few positive markers remains a complicated task.

Moreover, we observe low to medium expression of our candidates in close cell types from the same embryonic origin such as muscular cells and SMCs. This could be due to a shared phenotype between cells with close embryonic origin. Common markers between MSCs and SMCs have already been described. Notably, MSCs can express similar levels of SMC markers such as alpha-actin [90, 91]. Moreover, Kumar et al. [92] determined that MSCs, pericytes and SMCs could have the same mesenchymo-angioblast progenitor and that SMCs share a certain plasticity with MSCs, as they can be differentiated in chondrocyte-like and beige adipocytes or myo-fibroblasts. However, a lot of cell types in ENCODE have not been actively sorted by expression of their respective surface markers and fibroblast contamination is a classical feature in primary cell culture. Therefore, we should not exclude the possibility of fibroblast contamination when investigating markers for MSCs by bulk omics technology. Given this, scRNAseq could be the best solution to identify the source of marker expression in counterpart cells.

To conclude, our extensive cell type comparison shows that the discovery of a marker of MSCs as distinct cell type is not plausible. After deepening our own research on MSCs biomarkers at the annotated and unannotated levels, we were unable to find a marker that could simultaneously i) distinguish MSCs to close or homologous cell types (fibroblasts, satellite cells, SMCs), ii) be present in all MSCs types and iii) distinguish MSCs from more characterised cell types (hematopoietic lineage, neurones, etc). Our results suggest, like other studies, a strong proximity between MSCs, fibroblast and mesodermal cell types.

More than a marker of MSCs, candidates extracted by our method could be used to explore important features in MSCs biology and therefore, warrant investigation into their function, assuming that the specificity of RNA for a cell type can highlight its importance in cell activity. Even if the functional invalidation stands as the principal method to efficiently determine the function of a lncRNA, its expression and co-expression with known genes can potentially characterise a function or an intrinsic state of a cell type, particularly for MSCs with reported diversity of states and functions (differentiation, immunomodulation, senescence, proliferation, etc). In our opinion, it is vital that during the creation of a catalogue of lncRNAs, a restricted set of selected biomarkers should be studied more intensively, both in term of specificity and function. Assumptions on functional domains, where lncRNAs could act, could increase the relevance and visibility of discovered lncRNAs, and far from the bioinformatics implications, encourage future biological investigations. We decided to investigate the 3 selected Mlincs, validated by k-mers search, RT-qPCR and long-read sequencing, in term of biological impact with complementary in silico experimental approaches. We propose different in silico strategies, depending on the amount and diversity of the available data. The analysis confirms the non-coding potential of candidates and indicates a low probability of “miRNA sponge” activity. However, protein potential interaction results give interesting paths that were then investigated by complementary exploration. The k-mers quantification permits a naive high throughput exploration of numerous RNAseq data, simultaneously exploring potential functions and specificity to assess their potential. Instead of different cells, each candidate’s expression was quantified in MSCs in different experimental conditions. FANTOM6 data recently offered a pilot about lncRNAs functional investigation, with a high-throughput invalidation of 154 lncRNAs and coding genes in fibroblasts and their RNAseq counterpart added to phenotypical observations. The utilisation of co-expressions between KO genes and candidates lncRNAs remains an efficient way to decipher lncRNAs function, provided number of KD genes is high. Moreover, the availability of recent single-cell data of MSCs has been a good complement to lncRNAs functional investigation.

Using scRNAseq from Ad-MSCs [93], we observed that our markers are not expressed in all cells but constitute different subpopulations with different levels of rarity in Ad-MSCs. FANTOM6 and single-cell analysis could permit tracing three components of these states: stress inducible cells, lineage commited osteogenic progenitors and proliferating cells. Globally, we observed a global concordance of the results between the different strategies used for functional prediction. Mlinc.28428 has concomitant expression with genes related to the stress response pathway. Mlinc.28428 could be a good target for treatment to study the senescence process, age pathologies or stress response. Mlinc.128022 potentially interacts with THY1 (CD90) and has co-occurences with genes linked to osteoprogenitors and cell differentiation. The k-mers search highlights its participation in MSCs’ osteodifferentiation. Finally, Mlinc.89912 potentially interacts with damage repair and RNA decay, and tubulin metabolism, all linked to cell proliferation and cell cycle. Moreover, the subcompartment enrichment corresponds to this prediction: Mlinc.89912.1 is the only candidate to have possible interactions with DNA-repair system, a hypothesis corresponding to its observed enrichment in the nucleus. A final selection of bulk RNAseq of MSCs in specific biological conditions allowed confirmation of our initial assumptions, showing that the different strategies we propose could be used to give relevant indications of the lncRNAs’ functions. These results show that a lncRNA selected by its expression specificity has a high probability of being part of a functional mechanism.

## Conclusion

In conclusion, we have predicted genes and lncRNAs enriched in MSCs and proposed several selection steps including feature selection (machine learning), large scale signature search, RT-qPCR validation, in silico tools and single-cell analysis. We present the application of a new way of quantification in RNAseq: the specific k-mers search could be used as a naive information in lncRNAs catalogue creation. The strategies presented here are transferable to other cell types and different studies while the specificity and functional assumption present a significant potential in long non-coding transcriptome exploration. We present 3 lncRNA markers of bone marrow and adipose MSCs that passed all selection steps and present interesting features: Mlinc.28428.2, Mlinc.128022.2 and Mlinc.89912.1. These markers could be used by the scientific community as potential targets for functional biological experiments on MSCs, with pre-indications of potential functions to orientate the experiments and finally initiate the objective of transition between bioinformatics challenges and cell biology.

## Methods

### Data collection and basic processing

The public RNAseq datasets (in FASTQ format) have been assessed using ENCODE, the EBI “ArrayExpress” service or SRA database at each step of the pipeline: i) lncRNAs prediction and first differential analysis (Additional file 1), ii) k-mer search in ENCODE data to refine lncRNAs’ specificity (Additional file 8), iii) k-mer search in FANTOM6 CAGE dataset and scRNAseq analysis from Adipose MSCs by X. Liu *et al* raw data [93] for functional investigations (Additional files 15 and 16), iv) k-mer search in MSCs in different conditions (Additional file 18).

The reads quality were assessed with FastQC (https://www.bioinformatics.babraham.ac.uk/projects/fastqc/) to avoid the implementation of poor quality data in the analysis. Data from Peffers et al. [94], added to ENCODE’s BM-MSCs RNAseq data, were selected for the Mlinc and Mloanc characterisation and the differential analysis considering the above-mentioned features: Ribo-zero technology, stranded and paired-ends RNAseq. Peffers’ data had a forward-reverse library orientation instead of a reverse-forward orientation of a classic Illumina sequencing, thereby the order of paired files was manually reversed. To minimize false negative results in our analysis, we followed the standard ENCODE procedure which implies datasets with a minimum of ∼20*M* reads and we favored the use of ribodepletion method of extraction (details provided in Additional file 1). A single exception was made for hematopoietic progenitors (4 samples with ∼5*M* reads and 2 other ones with ∼25*M* and ∼30*M* reads), justified by the lack of public data and the relevance of a comparison hematopoietic/mesenchymal cells. The FASTQ files used for lncRNAs prediction in MSCs referred as “MSC” group (Additional file 1), were mapped using CRAC v2.5.0 software [95] on the indexed GRCh38 human genome including mitochondria, with –stranded, -k 22 and –rf options.

### Ab initio assembly for transcripts prediction or unannotated transcripts prediction

The aligned reads of the “MSC” group were put through ab initio transcript assembly. Unannotated transcripts were predicted with the following procedure: i) an ab initio reconstruction was performed on individual RNAseq with StringTie [96] version 1.3.3b, with -c 5 -j 5 rf -f 0.1 options (5 spliced reads are necessary to predict a junction and a minimum of 5 reads are required to predict an expressed locus), ii) the output individual GTF files obtained with the RNAseq of “MSC” group were then merged with StringTie with -f 0.01 -m 200 options and with a minimum TPM of 0.5, with the Ensembl human annotation (GRCh38) v90 used as guide for StringTie. The GTF was parsed with BEDTools [97] to dissociate new intergenic lncRNAs (lincRNAs) from annotated RNAs (coding or annotated lncRNAs), by applying filter criteria classically used in lncRNAs prediction [98], excluding transcript models overlapping (by 1 bp or more) any annotated coordinates. The resulting GTF of unannotated lincRNAs from MSCs is referred as “Mlinc”. In parallel, the GTF was parsed with BEDTools to dissociate overlapping-antisens lncRNAs (lncoaRNAs), by applying filter criteria classically used in lncRNAs prediction, keeping transcript overlapping any annotated coordinates, then excluding transcripts overlapping these annotated coordinates on the same strand. The resulting GTF of MSCs overlapping-antisens lncRNAs is referred as “Mloanc” (Fig. 1). For an exhaustive analysis, we decided not to filter the reconstructed transcripts by their mono-exonic structure but selected ab initio reconstructions bigger than 200 bp. Potential false positives can later be eliminated in the downstream steps such as differential expression analysis, long-read sequencing and qPCR.

### Long-read sequencing

The library was generated with 250 ng polyA+ mRNA purified from 50 *μ*g of human BM-MSCs total RNA. The polyA+ mRNAs were treated according to the cDNA-PCR sequencing kit protocol (ref SQK-PCS108) as recommended by ONT. 3 254 396 sequences were obtained on the ONT MinION sequencer. The base calling was done with albacore version 2.2.7. 2 720 928 long reads were successfully mapped using Minimap2 [99] version 2.10-r764 on GRCh38 human genome with default options used for ONT sequencing.

### Quantification with pseudoalignment and feature selection

Kallisto v0.43.1 [23] was used directly on RNAseq raw FASTQ from the “MSC” and “non-MSC” groups. This pseudoalignment was performed with a number of bootstraps (-b) of 100, using a Kallisto index containing the sequences of all transcripts: the Ensembl coding and non-coding transcripts (v90) plus the predicted lincRNAs and lncoaRNAs. Sleuth version 0.29.0 [24] was used with R for differential expression analysis using the Wald test method, to compare the “MSC” group against the “non-MSC” group (including lymphocytes, macrophages, hepatocytes, iPSCs, ESCs, HUVECs, neurons, chondrocytes). Analysis was performed at the gene level for the annotated genes and at the transcript level for the predicted lincRNAs and lncoaRNAs. Genes or lncRNAs having a log2 FC between “MSC” and others greater than 0.5 and a *p*-value lower than or equal to 0.05 were selected. Finally, only transcripts/genes overexpressed in MSCs were selected. Each category (annotated transcripts, lincRNAs and lncoaRNAs) of potential candidates passing the first differentiation expression filter were separated for feature selection analysis. Boruta 6.0 [29] was used with 10000 maximum runs and a *p*-value of 0.01 on each category, with multiple comparisons adjustment using the Bonferroni method (mcAdj = TRUE). Candidates passing the Boruta test as “Confirmed” for each category were selected as reliable biomarkers.

### Quantification by k-mers search

To quantify the expression of a transcript or a gene in available RNAseq data with a rapid procedure, specific 31nt long k-mers were extracted from the candidate sequences. A specific k-mer of an annotated candidate corresponds to a 31nt sequence that maps once on the genome and reference transcriptome (Ensembl v90). In case of unannotated transcript (Mlinc, Mloanc), a specific k-mer maps once on the genome and is absent from the reference transcriptome. The automated selection of specific k-mers is ensured by the Kmerator tool (manuscript in preparation, https://github.com/Transipedia/kmerator). The k-mers were then quantified directly in raw FASTQ files using countTags (https://github.com/Transipedia/countTags). The quantification is expressed by the average count of all k-mers for one transcript, normalised by million of total k-mers in the raw file.

In FANTOM6 Dataset (Additional file 1510.1101/700864) containing CAGE analysis, to approach a TPM normalisation, the number of k-mers quantified was normalised by the total number of reads in million.

### Genomic intervals assessment

DNase-seq intervals of enrichment were directly downloaded from ENCODE in bed format for BM-mesenchymal cells (ENCFF832FHZ) and hematopoietic progenitors (ENCFF378FCS). The H3K27ac (GSM3564514) and H3K4me3 (GSM3564510) ChIP results from undifferentiated BM-MSCs of the Agrawal Singh S. et al. study [82] were downloaded from GEO database in WIG format, and remapped to the GRCh38 genome with CrossMap (http://crossmap.sourceforge.net/). PolyA+ CAGE localisations from ENCODE/RIKEN were downloaded in.bed format from UCSC Table Browser with “GRCh37” assembly and “Expression” group (“TSSHMM” files at: https://genome.ucsc.edu/cgi-bin/hgTables). The downloaded files corresponding to samples of MSCs from BM, Ad and UC (named hMBM, hMAT,and hMUC respectively), CD34 and H1ES cells were then remapped to the GRCh38 genome with liftOver (https://genome.ucsc.edu/util.html).

### In silico functional prediction

We used LncADeep [33] to identify particular correlations between candidates and proteins. Beginning with our selection of 3 candidates, we filtered shared predicted proteins and selected proteins predicted as interacting uniquely with the concerned candidate. The pathways concerned with these unique proteins were identified with Reactome. TarpMir was used to identify possible target sites of human miRNA from miRbase (*p*=0.5) [32] and FEELnc [31] to decipher the coding potential of candidates, using the coding and non-coding part of Ensembl annotation sequences as model.

### Single-cell analysis

Single-cell data were pseudoaligned with Kallisto, with the same index used for the initial bulk RNAseq analysis. Pseudoalignment of 10X genomics data, correction, sorting and counting were made by Kallisto “bus” function. Count matrices were processed with Seurat R package [100*,*^101^]. Empty droplets were estimated by barcode ranking knee and inflection points, only droplet with a minimal count of 10000 were kept. In the end, 26071 droplets remain. After normalisation, Inter-donor batch effect was corrected with ComBat method in sva R package [102] (Combat function, prior.plots=FALSE, par.prior=TRUE). Cell cycle scoring was made by CellCycleScoring Seurat function, using gene set used by the initial authors [93]. Finally, other sources of unnecessary variability as percent of mitochondrial genes, cell cycle and number of unique molecular identifiers (UMIs) were regressed using ScaleData Seurat function.

To decipher genes enriched in cells positive for our markers, cells with a scaled expression superior or equal to 0.1 were labelled as positive, whereas cells with an expression inferior to the level were labelled as negative. Then, markers of these cells were deciphered using FindAllMarkers Seurat function with a minimum FC threshold of 0.15. Expression of our markers in the Ad-MSCs population was made by FeaturePlot Seurat function after UMAP dimensional reduction, the gene enrichments were represented with VlnPlot function.

### Data visualisation

Genome browser-like figures were generated with Gviz R package [103]. BAM tracks were generated from merged BAM files used for transcript prediction. Heatmaps were generated using superHeat R package (https://github.com/rlbarter/superheat).

### Cell preparation and culture conditions

MSCs were isolated from bone marrow aspirates of patients undergoing hip replacement surgery, as previously described [104]. Cell suspensions were plated in *α*-MEM supplemented with 10% FCS, 1 ng/mL FGF2 (R&D Systems), 2 mM L-glutamine, 100 U/mL penicillin and 100 *μ*g/mL streptomycin. These were shown to be positive for CD44, CD73, CD90 and CD105 and negative for CD14, CD34 and CD45 and used at the third or fourth passage. Human skin fibroblasts were cultured in DMEM high glucose supplemented with 10% FCS. For Ad-MSCs isolation, adipose tissue was digested with 250 U/mL collagenase type II for 1 h at 37 ^∘^C and centrifuged (300 g for 10 min) using routine laboratory practices. The stroma vascular fraction was collected and cells filtered successively through a 100 *μ*m, 70 *μ*m and 40 *μ*m porous membrane (Cell Strainer, BD-Biosciences, Le-Pont-de-Claix, France). Single cells were seeded at the initial density of 4000 cell/cm2 in *α*MEM supplemented with 100 U/mL penicillin/streptomycin (PS), 2 mmol/mL glutamine (Glu) and 10% fetal calf serum. After 24 h, cultures were washed twice with PBS. After 1 week, cells were trypsinised and expanded at 2000 cells/cm2 till day 14 (end of passage 1), where Ad-MSCs preparations were used.

HUVECs obtained from Clonetics (Lonza, Levallois Perret, France) were cultured in complete EGM-2MV (Lonza) supplemented with 3% FCS (HyClone; Perbio Science, Brebières, France).

Primary human myoblasts were isolated and purified from skeletal muscles of donors, as described by Kitzmann *et al* [105]. Purified myoblasts were plated in Petri dishes and cultured in growth medium containing Dulbecco’s Modified Eagle’s Medium (Gibco) supplemented with 20% foetal bovine serum (GE Healthcare, PAA), 0.5% Ultroser G serum substitute (PALL life sciences) and 50 *μ*g/ml Gentamicin (Thermo Scientific, France) at 37 ^∘^ C in humidified atmosphere with 5% CO2. All experiments were carried out between passage 4 (P4) and P8 to avoid cell senescence.

IPSCs were maintained in mTeSR-1TM medium (STEMCELL Technologies), in Petri dishes with matrigel (Corning, France). For the passages, cells were incubated in Gentle Cell Dissociation Reagent (STEMCELL Technologies) at room temperature, dissociation medium was discarded and cells incubated in mTeSR medium. All cell cultures were performed at 37 ^∘^C with 5% of O2 and 10% of CO2.

Primary human hepatocytes (PHHs) were isolated, as described previously [106], from liver resections performed in adult patients.

NSC derived from H9 or directly bought (StemPro) have been cultivated on laminine with StemPro NSC SFM medium.

H9 ESCs were cultivated in ESICO medium in a coculture H9/MEF (Mouse Embryonic Fibroblasts) at 37 ^∘^C with 5% of O2 and 5% CO2.

### RNA preparation and reverse transcription

Total RNA was isolated using TRIzol reagent (invitrogen) or RNeasy Mini Kit (Qiagen, France) according to the manufacturer protocol. RNA was quantified using a NanoDrop ND-1000 spectrophotometer (Thermo Fisher Scientific, France). RNA quality and quantity were further assessed using the 2100-Bioanalyzer (Agilent Technologies, Waldronn, Germany). Only preparations with RNA integrity number (RIN) values above 7 were considered. Reverse-transcription was performed either with random hexamers using the GeneAmp Gold RNA PCR Core kit (Applied Biosystems) or with oligo(dT) using SuperScriptTM First-Strand Synthesis System for RT-qPCR (invitrogen, France).

### Real-time quantitative PCR

Primer pairs were designed with primer3 online software (http://bioinfo.ut.ee/primer3-0.4.0/) from the transcripts’ sequences. Primer pairs with a perfect and unique match on the human genome were validated with UCSC blat software (https://genome.ucsc.edu). As a final verification, primers were visualised in parallel with the BAM alignment using IGV (http://software.broadinstitute.org/software/igv/) to verify that the primers overlap zones with read coverage. If possible, primer-pairs were designed to span an intron when present in the genomic sequence. Primers were designed for a mean Tm of 60 ^∘^C. Quantitative PCR (qPCR) were performed using LightCycler 480 SYBR Green I Master mix and real-time PCR instrument (Roche). PCR conditions were 95 ^∘^C for 5 min followed by 45 cycles of 15 s at 95 ^∘^C, 10 s at 60 ^∘^C and 20 s at 72 ^∘^C. For each reaction, a single amplicon with the expected melting temperature was obtained.

The gene encoding ribosomal protein S9 (RPS9) was used as house-keeping gene for normalisation. The cycle threshold (Ct) of each amplification curve was calculated by Roche’s LightCycler 480 software using the second derivative maximum method. The relative amount of transcripts were calculated using the ddCt method [107].

## Supplementary Information

**Additional file 1** Details and metadata of RNAseq dataset(s) downloaded for unannotated lncRNA prediction and differential expression between “MSC” and “non-MSC” groups.

**Additional file 2** List of differentially expressed annotated genes between “MSC” and “non MSC” groups.

**Additional file 3** Expression of Mloancs (antisens unannotated lncRNAs) selected after feature selection in the differential analysis cohort.

**Additional file 4** All information needed about selected Mlincs (differentially expressed and selected by Boruta feature selection).

**Additional file 5** Expression of ISCT’s MSC markers in the differential analysis cohort; THY1 = CD90, NT5E = CD73, ENG = CD105, ITGAM = CD11B, PTPRC = CD45.

**Additional file 6** Heatmap presenting positive markers for MSC proposed in the bibliography.

**Additional file 7** CAGE enrichment sites for predicted Mlincs. Genomic visualisation of Mlincs 28428 (top left panel), 64225 (top right panel), 128022 (bottom left panel), and 89912 (bottom right panel). For each panel, genomic position is presented on the top. Predicted Mlincs (orange) are compared to non-oriented long-read alignments (grey). Below, black arrows represent PolyA (PA+) CAGE enrichment sites in MSC from Adipose tissue (Ad-MSC), Umbilical Cord (UC-MSC) and Bone Marrow (BM-MSC) and are compared to H1 Embryonic Stem cells and CD34 cells. CAGE data collected from UCSC Table browser (see “Methods” section).

**Additional file 8** Details and metadata of RNAseq dataset downloaded from ENCODE for k-mer based quantification of candidates in diversified types of cells.

**Additional file 9** Relative expression of the positive marker ENG (CD105) across ENCODE’s ribodepleted RNAseq data, made by k-mer quantification, normalised in k-mer by million.

**Additional file 10** Relative expression of the positive marker NT5E (CD73) across ENCODE’s ribodepleted RNAseq data, made by k-mer quantification, normalised in k-mer by million.

**Additional file 11** Relative expression of the positive marker of THY1 (CD90) across ENCODE ribodepleted RNAseq data, made by k-mer quantification, normalised in k-mer by million.

**Additional file 12** Relative expression of the positive markers Mlinc.64225.1 across ENCODE’s ribodepleted RNAseq data, made by k-mer quantification, normalised in k-mer by million.

**Additional file 13** Primer position on selected Mlinc candidates and corresponding expression in MSCs, HUVECs and myoblasts.

**Additional file 14** Potential interactions between selected Mlincs and annotated proteins, found by LncADeep.

**Additional file 15** Details and metadata of FANTOM6 dataset used for functional prediction.

**Additional file 16** Single-cell RNAseq data of Adipose MSCs by X. Liu et al, used for co-expression research at the cell level.

**Additional file 17** Distribution of cycle phases between Mlinc-positive and Mlinc-negative MSCs at single cell level.

**Additional file 18** Details and metadata of RNAseq dataset downloaded for k-mer based quantification of candidates in MSCs in different biological situations.

## Data Availability

The data for this study have been deposited in the European Nucleotide Archive (ENA) at EMBL-EBI under accession number PRJEB41537 (https://www.ebi.ac.uk/ena/browser/view/PRJEB41537). All RNAseq and FANTOM6 CAGE data analysed during this study are available in SRA database (https://www.ncbi.nlm.nih.gov/sra), or ENCODE database (https://www.encodeproject.org/search/?type=Experiment&status=released&perturbed=false). The corresponding references and associated databases are specified in this article and its supplementary information files 1, 2, 3, 5, 6, 8, 15, 16 and 16. WIG files used to assess methylation and acetylation of chromatin at the Mlincs sites are directly downloaded from Gene Expression Omnibus (https://www.ncbi.nlm.nih.gov/geo/). The human genome and transcriptome sequences and intervals (coding and non-coding) used as references are available in Ensembl site (https://www.ensembl.org/info/data/ftp/index.html).

## Abbreviations

Ad: Adipose tissue
BM: Bone marrow
CAGE: Cap analysis of gene expression
Ct: Cycle threshold
CytoD: Cytochalasin D
ENG: Endoglin
FC: Fold change
hESC: Human embryonic stem cell
HUVEC: Human umbilical vein endothelial cell
ISCT: International society for cellular therapy
iPSC: Induced pluripotent stem cell
KD: Known-down
KO: Knock-out
lncRNA: long non-coding RNA
MEF: Mouse embryonic fibroblast
Mlinc RNA: MSC-related long intergenic non-coding RNA
Mloanc RNA: MSC-related long overlapping antisense non-coding RNA
MSC: Mesenchymal stem cell
NMD: Nonsense mediated decay
NHEJ: Non-homologous end-joining
ONT: Oxford nanopore technologies
PHH: Primary human hepatocytes
RNAseq: RNA sequencing
RT-qPCR: Real-time quantitative PCR
scRNAseq: single-cell RNAseq
SMC: Smooth muscle cell
TPM: Transcripts per million
TPM2: Tropomyosin 2
UBB: Ubiquitin B
UC: Umbilical cord
UMI: Unique molecular identifier
