# Supplementary figures and images for: Long non-coding RNA exploration for mesenchymal stem cell characterisation
Source: BMC Genomics. 2021 Jun 4;22:412. doi: 10.1186/s12864-020-07289-0 (PMC8178833; doi:10.1186/s12864-020-07289-0)

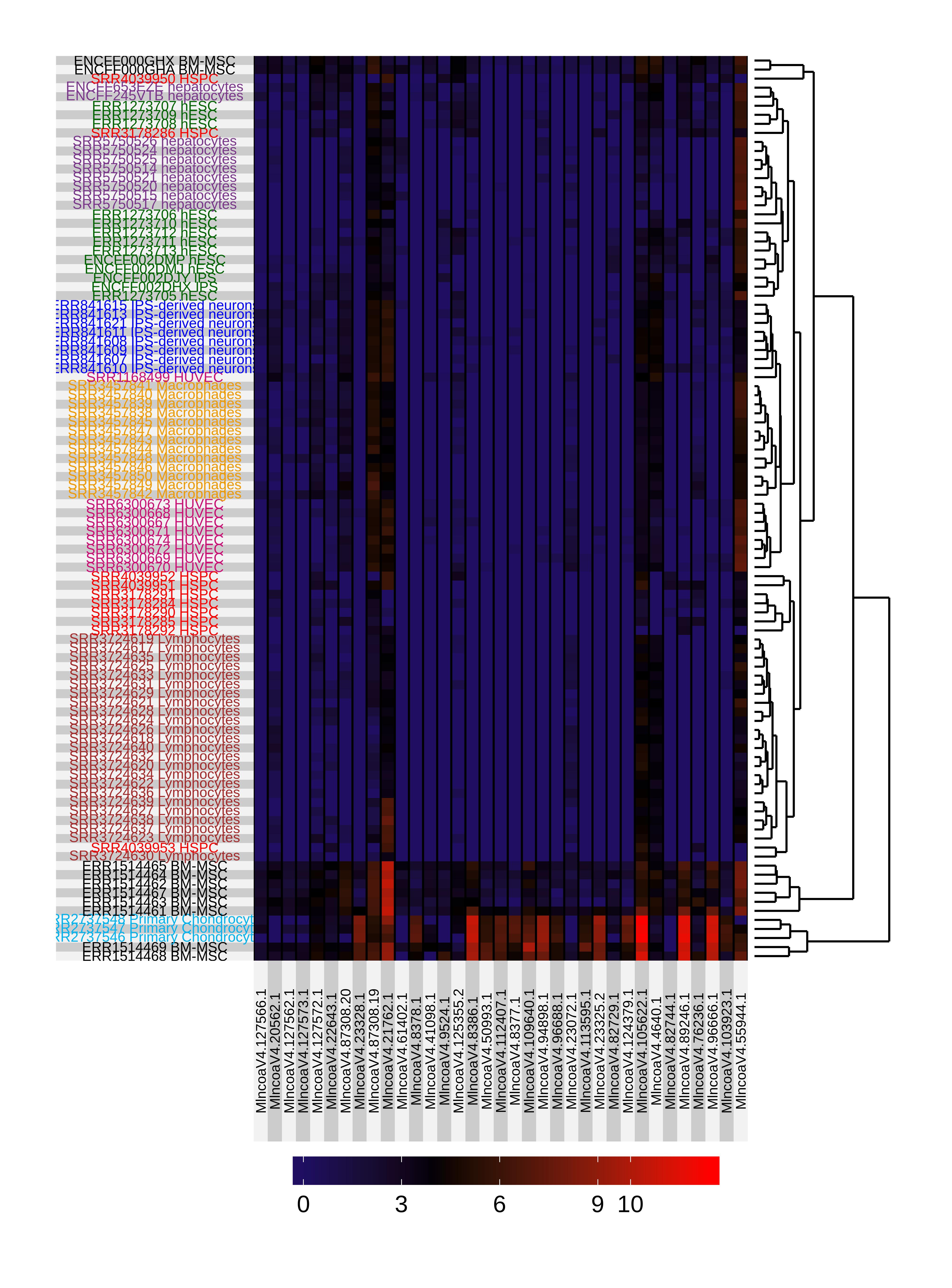

Supplement: Supplementary file 3 — Additional file 3 Expression of Mloancs (antisens unannotated lncRNAs) selected after feature selection in the differential analysis cohort. [file 12864_2020_7289_MOESM3_ESM.jpeg]

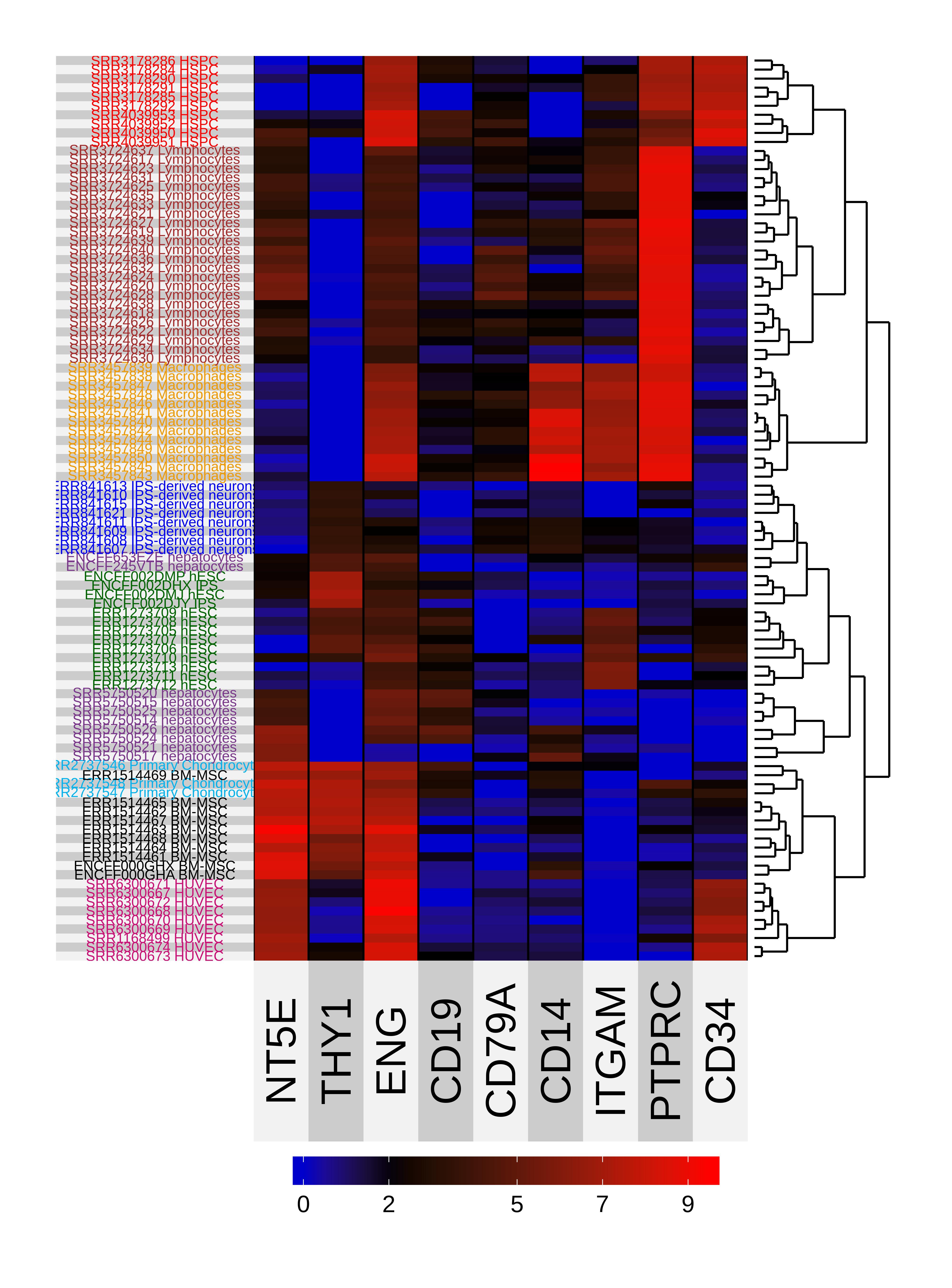

Supplement: Supplementary file 5 — Additional file 5 Expression of ISCT’s MSC markers in the differential analysis cohort; THY1 = CD90, NT5E = CD73, ENG = CD105, ITGAM = CD11B, PTPRC = CD45. [file 12864_2020_7289_MOESM5_ESM.jpeg]

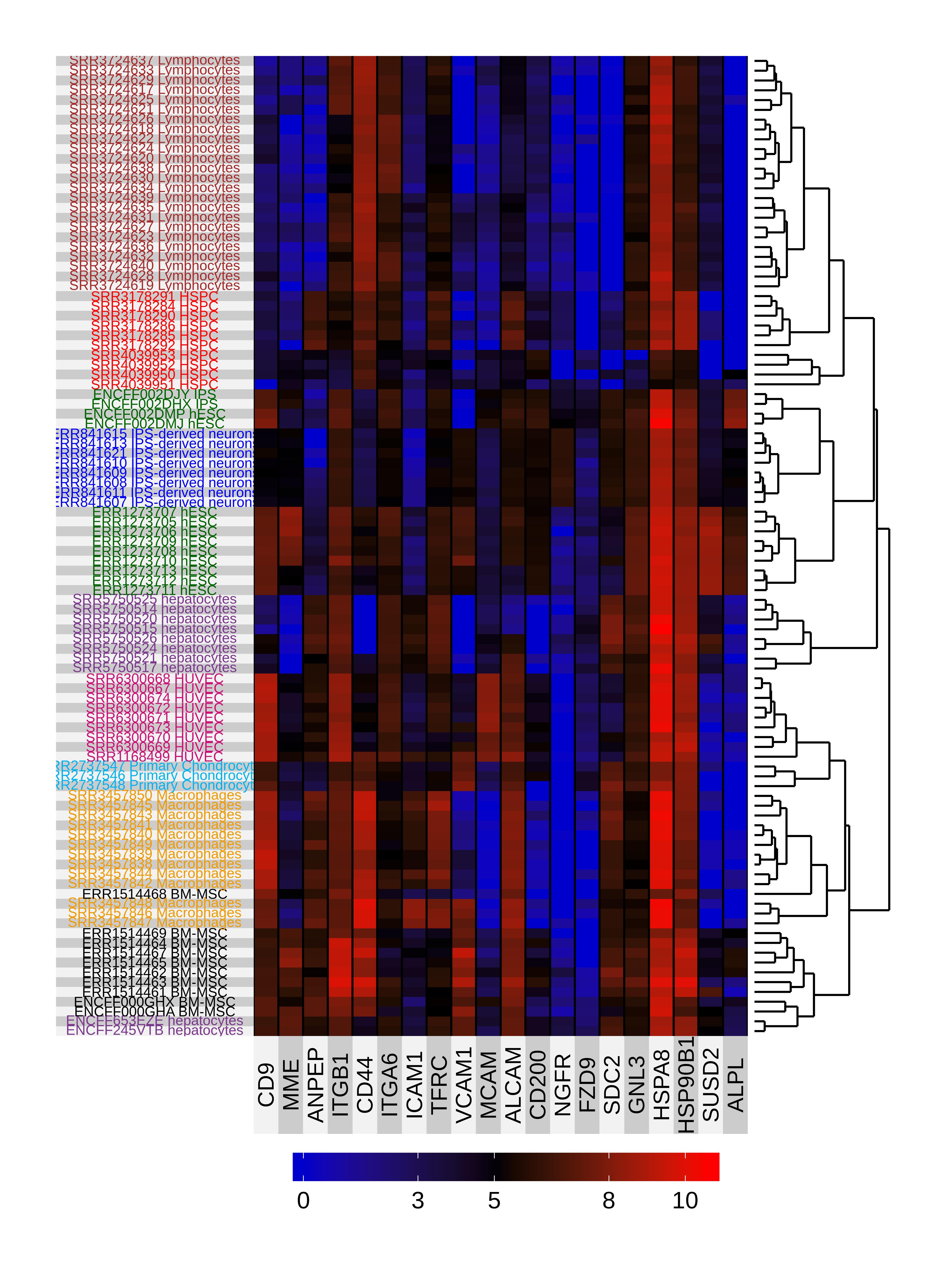

Supplement: Supplementary file 6 — Additional file 6 Heatmap presenting positive markers for MSC proposed in the bibliography. [file 12864_2020_7289_MOESM6_ESM.jpeg]

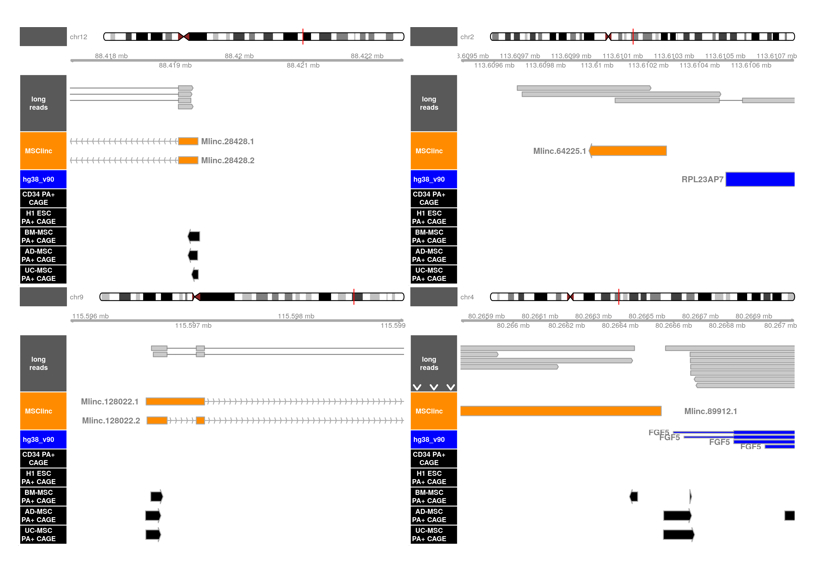

Supplement: Supplementary file 7 — Additional file 7 CAGE enrichment sites for predicted Mlincs. Genomic visualisation of Mlincs 28428 (top left panel), 64225 (top right panel), 128022 (bottom left panel), and 89912 (bottom right panel). For each panel, genomic position is presented on the top. Predicted Mlincs (orange) are compared to non-oriented long-read alignments (grey). Below, black arrows represent PolyA (PA+) CAGE enrichment sites in MSC from Adipose tissue (Ad-MSC), Umbilical Cord (UC-MSC) and Bone Marrow (BM-MSC) and are compared to H1 Embryonic Stem cells and CD34 cells. CAGE data collected from UCSC Table browser (see “Methods” section). [file 12864_2020_7289_MOESM7_ESM.jpg]

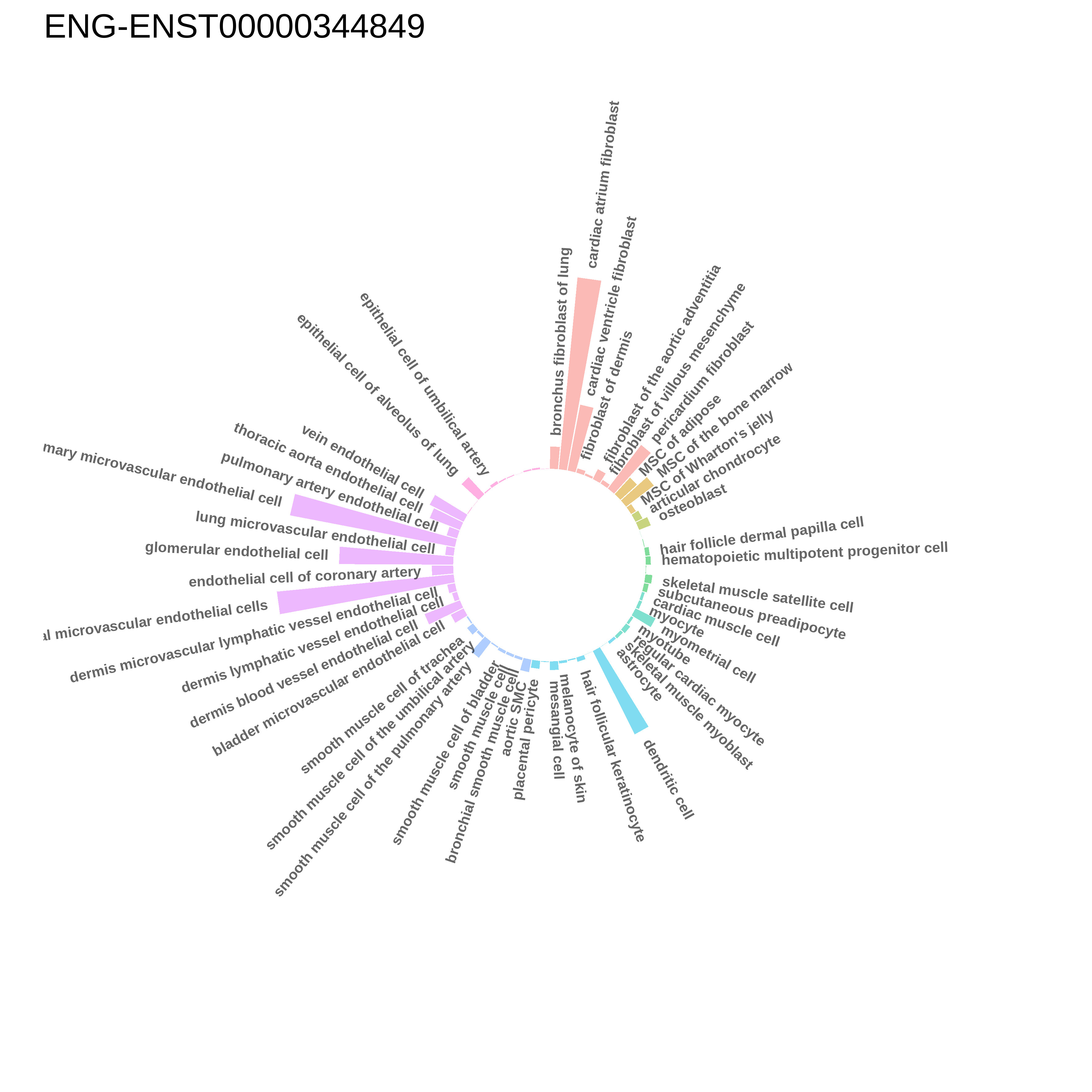

Supplement: Supplementary file 9 — Additional file 9 Relative expression of the positive marker ENG (CD105) across ENCODE’s ribodepleted RNAseq data, made by k-mer quantification, normalised in k-mer by million. [file 12864_2020_7289_MOESM9_ESM.jpg]

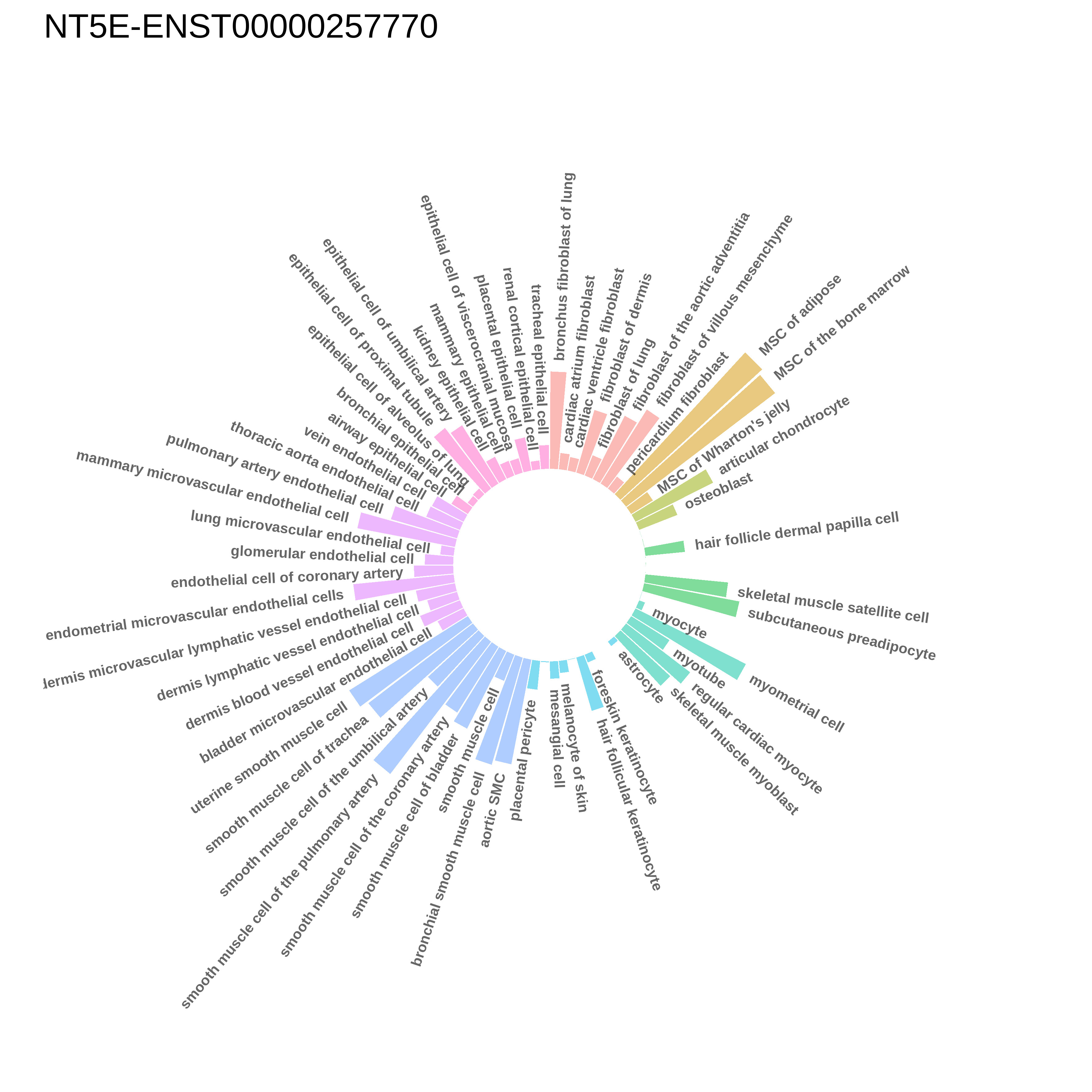

Supplement: Supplementary file 10 — Additional file 10 Relative expression of the positive marker NT5E (CD73) across ENCODE’s ribodepleted RNAseq data, made by k-mer quantification, normalised in k-mer by million. [file 12864_2020_7289_MOESM10_ESM.jpg]

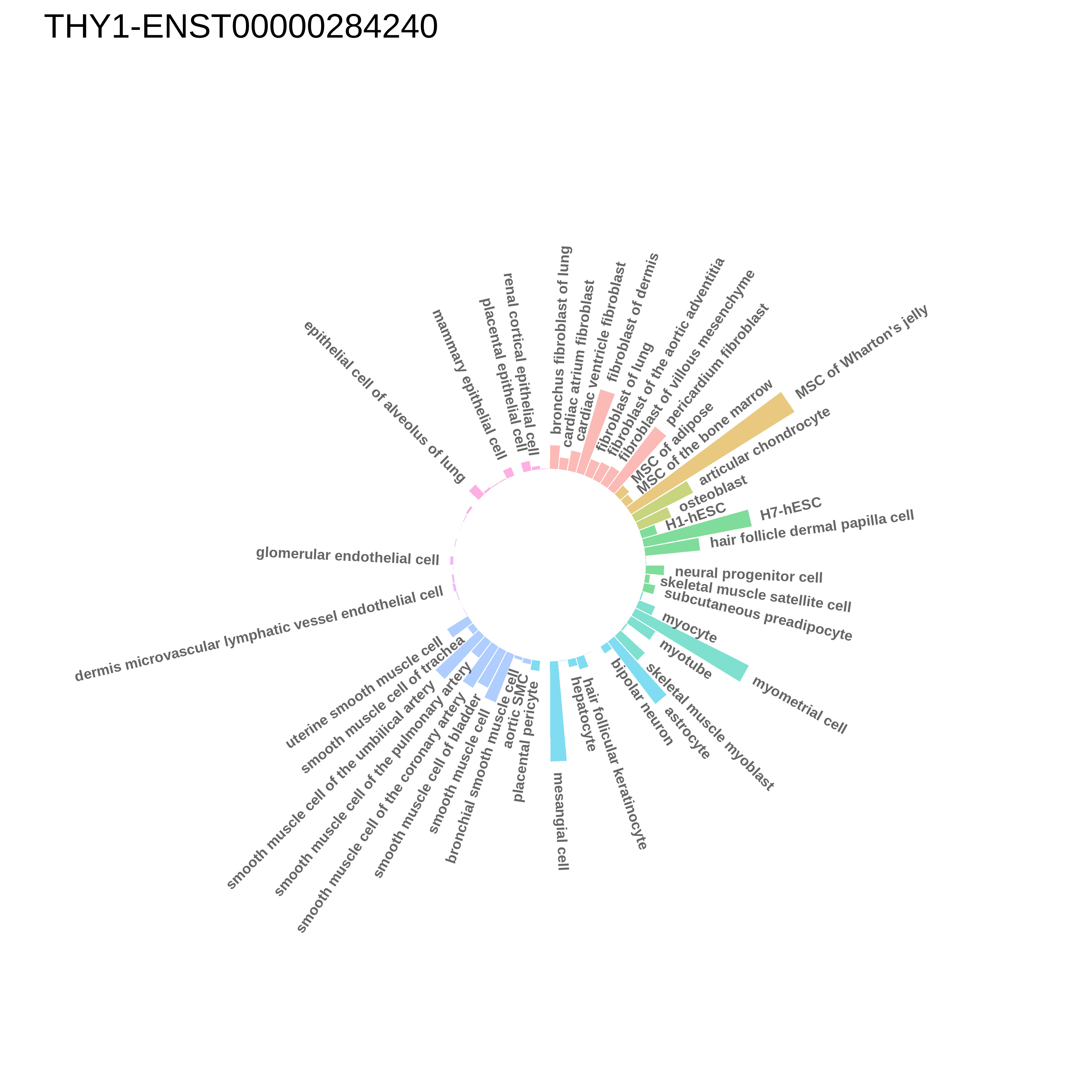

Supplement: Supplementary file 11 — Additional file 11 Relative expression of the positive marker of THY1 (CD90) across ENCODE ribodepleted RNAseq data, made by k-mer quantification, normalised in k-mer by million. [file 12864_2020_7289_MOESM11_ESM.jpg]

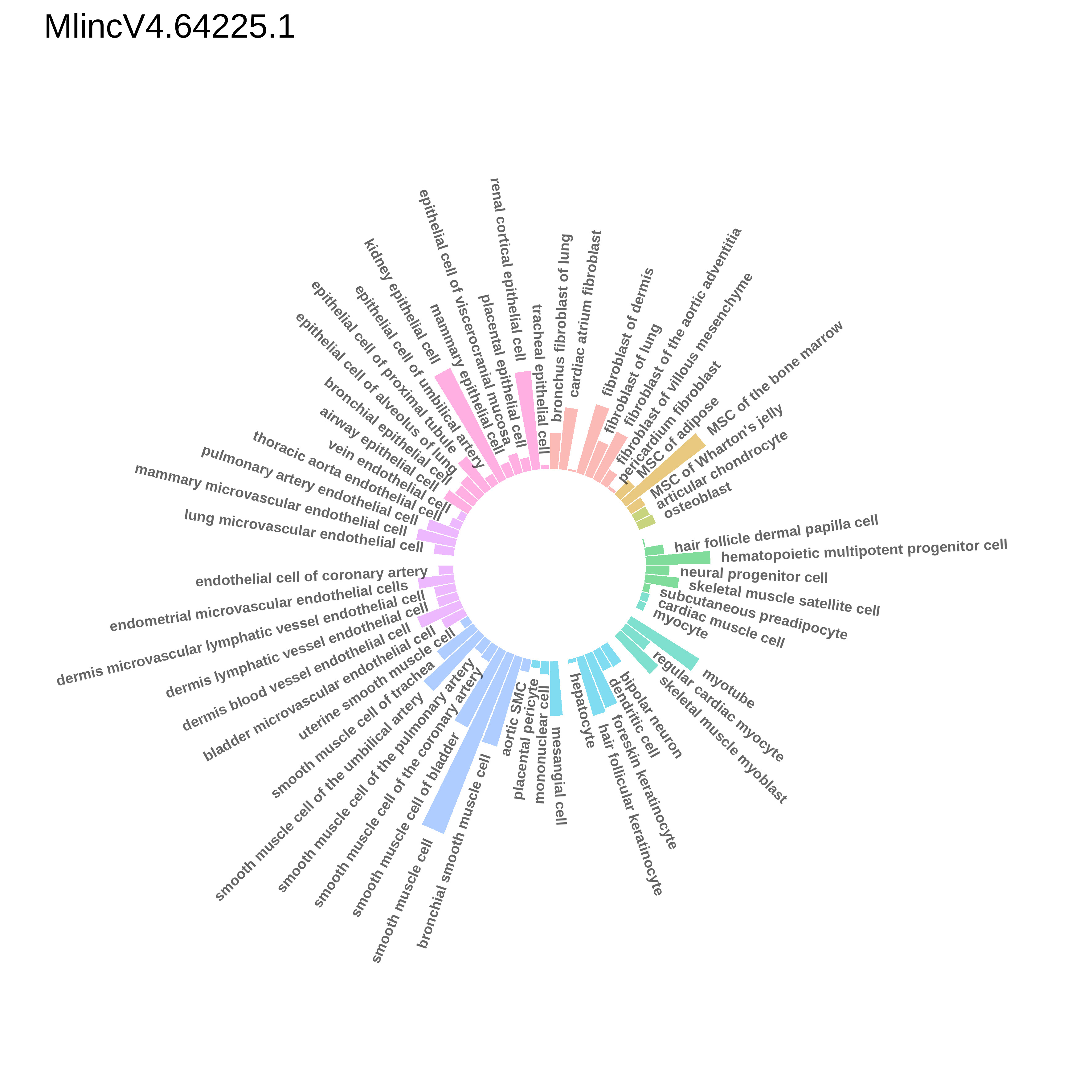

Supplement: Supplementary file 12 — Additional file 12 Relative expression of the positive markers Mlinc.64225.1 across ENCODE’s ribodepleted RNAseq data, made by k-mer quantification, normalised in k-mer by million. [file 12864_2020_7289_MOESM12_ESM.jpg]

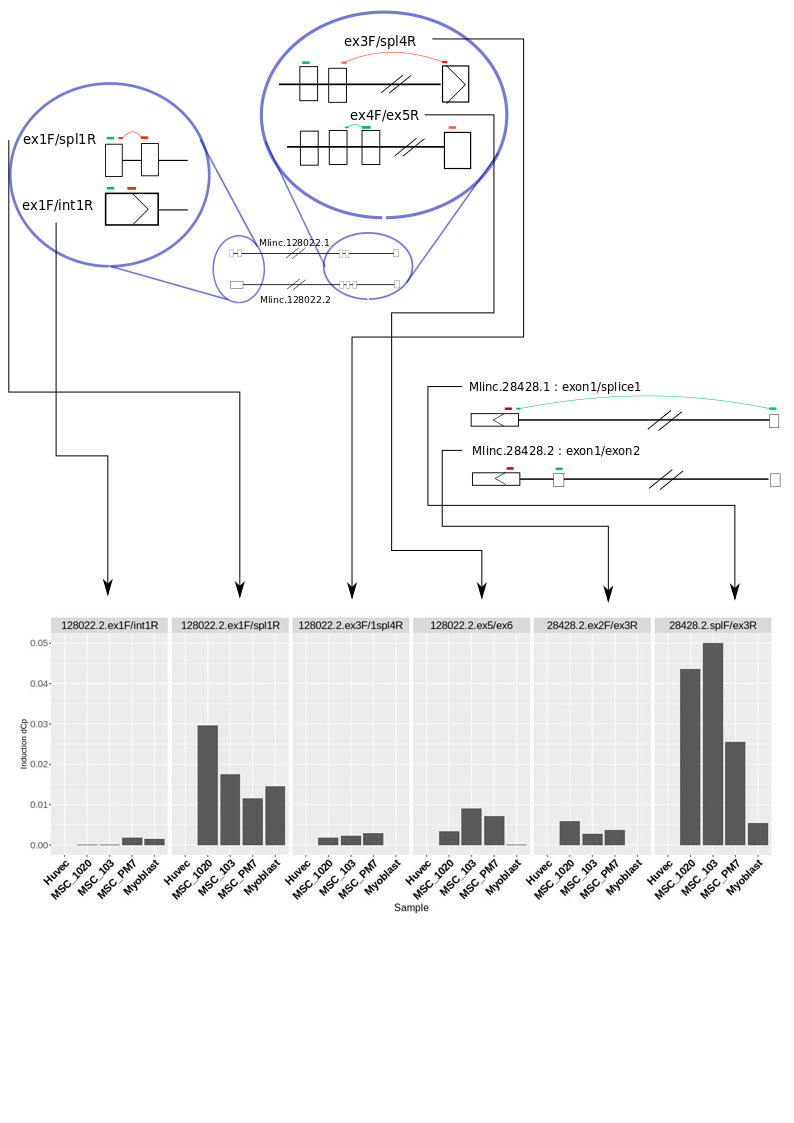

Supplement: Supplementary file 13 — Additional file 13 Primer position on selected Mlinc candidates and corresponding expression in MSCs, HUVECs and myoblasts. [file 12864_2020_7289_MOESM13_ESM.jpg]

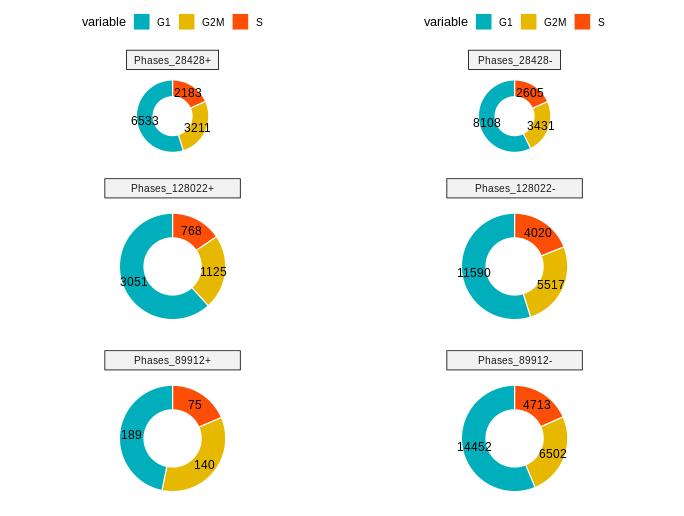

Supplement: Supplementary file 17 — Additional file 17 Distribution of cycle phases between Mlinc-positive and Mlinc-negative MSCs at single cell level. [file 12864_2020_7289_MOESM17_ESM.jpeg]
